# High-Precision Surface Tension Measurements of Sodium, Potassium, and Their Alloys via Du Noüy Ring Tensiometry

**DOI:** 10.1021/acsami.5c02183

**Published:** 2025-04-21

**Authors:** Naiyu Qi, Rachana Somaskandan, Gustav Graeber

**Affiliations:** † Graeber Lab for Energy Research Department of Chemistry, Humboldt-Universität zu Berlin, Berlin 12489, Germany; ‡ Department of Chemical Engineering, Northeastern University, Boston, Massachusetts 02115, United States; § Department 3: Containment Systems for Dangerous Goods; Energy Storage, Federal Institute for Materials Research and Testing, (BAM), Berlin 12205, Germany

**Keywords:** sodium−potassium alloy, liquid-metal anodes, alkali metal, batteries, surface tension, tensiometry

## Abstract

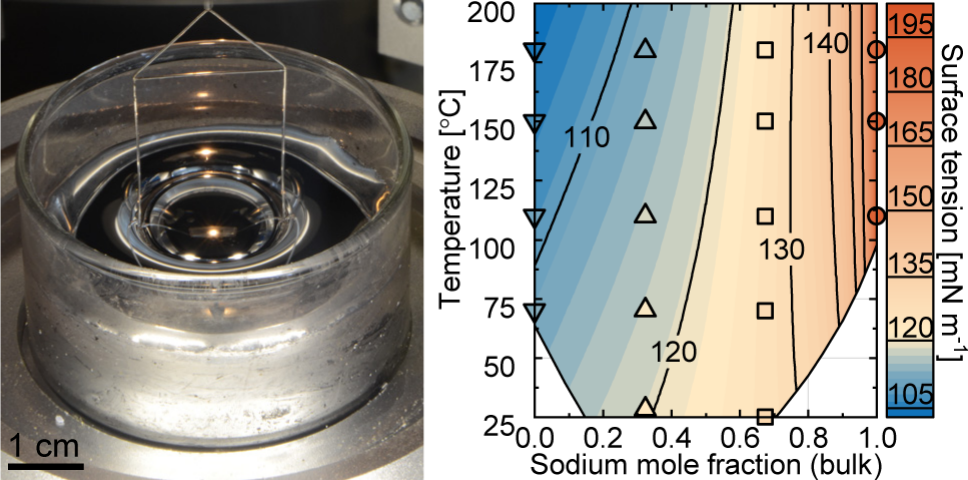

The development of post-lithium-ion batteries has sparked significant interest in alkali-metal anodes, particularly sodium (Na), potassium (K), and sodium–potassium (Na–K) alloys. Na–K alloys are promising for partially liquid anodes due to their unique low melting points. A critical factor influencing Na–K-based anode performance is wetting behavior, which governs electrical conductivity, mechanical contact, and long-term stability. At the heart of wetting lies surface tension, a fundamental property of solid–liquid–gas interactions. However, the surface tension of alkali metals and their alloys, particularly Na–K systems, remains poorly understood due to experimental and theoretical challenges. This study bridged these gaps by employing Du Noüy ring tensiometry for the first time in alkali-metal systems to measure the surface tension of Na, K, and Na–K alloys across temperatures from ambient to 180 °C. A key innovation in this work is the development of the push-in Du Noüy method, which provided significantly higher precision and reliability compared to the traditional pull-out technique, without requiring a correction factor. The measured surface tension decreased with increasing temperature for the studied Na–K alloys. For instance, for a eutectic Na–K mixture, the surface tension decreases from 121.7 mN m^–1^ to 112.2 mN m^–1^ when increasing the temperature from ambient to 180 °C. Additionally, this study presented the first use of Gibbs free energy minimization to model the surface tension of the Na–K system. The robust method significantly enhanced the predictive accuracy compared to the previous simplified model, reducing deviations from 25% to 2%. Our findings reveal that surface tension increases with sodium mole fraction in the bulk phase, yet the surface monolayer remains potassium-rich, indicating non-ideal surface behavior. This study deepens the understanding of alkali-metal wetting behavior, providing valuable insights for designing optimized interfaces in next-generation semi-solid alkali-metal batteries.

## Introduction

The global push for an energy transition is essential to combating climate change, conserving natural resources, and securing a sustainable future. Developing efficient and environmentally friendly energy storage systems has thus become a critical focus. While lithium-ion batteries currently dominate the market due to their established performance, their limitations including uneven global resource distributions, high costs, and substantial environmental impact during their life cycle necessitate the development of alternative solutions.^1−4^ Sodium (Na), potassium (K), and their alloys (Na–K) are emerging as compelling candidates for next-generation high-performance post-lithium-ion batteries.^5^ These materials offer advantages such as abundance, low cost, and favorable electrochemical properties, aligning well with the goals of a sustainable energy transition.^6−10^ Na–K alloys stand out due to their low melting points, which have made them widely used as coolants in experimental neutron nuclear reactors. Unlike sodium or lead coolants, which require continual heating to stay liquid in commercial reactors, the eutectic Na–K alloy remains liquid at room temperature. This eliminates the need for extra energy input and makes it ideal for fast reactors that are frequently shut down and defuelled.^11^ Beyond nuclear applications, researchers have also found Na–K’s great potential to operate at low temperatures with substantially enhanced electrochemical performance. The Na–K phase diagram shows a broad liquid-phase region between 15% and 70% mole fraction of sodium at room temperature, which can facilitate improved ion transport and mitigate challenges like dendrite formation when integrated with rational battery design.^12−16^ Albertus et al. suggested that substituting an intercalation-type anode with a pure alkali-metal electrode could increase specific energy by approximately 35% and energy density by around 50% at the cell level, highlighting the significant potential of alkali-metal anodes.^1^ Park et al. reported further advancements in Na–K alloy performance, demonstrating K^+^ critical current densities exceeding 15 mA cm^–2^ when paired with K-β″-Al_2_O_3_ electrolyte. Their research also showed that incorporating a Na–K liquid wetting interfacial film between lithium metal and a solid electrolyte doubled the critical current density and enabled cycling at impressive areal capacities over 3.5 mAh cm^–2^.^12^ Zhao et al. developed an oxygen-rich carbon fiber cloth that exhibited superwetting properties for Na–K liquid metal through an enthalpy-driven wetting process. This composite demonstrated cycling stability for over 1600 h and showed excellent flexibility for pouch cell applications, which could effectively inhibit dendrite growth due to the self-adaptive nature of the liquid metal. Yet, the pouch cell only retains 92.3% capacity after 40 cycles, indicating the need for further enhancement in long-term performance.^13^ Cheng et al. developed a carbon-fiber-supported liquid Na–K alloy anode that demonstrated exceptional performance in solid-state sodium metal batteries. Their cell achieved cycling stability over 800 h with an areal capacity of 30 mAh cm^–2^ while reaching a critical current density of 40 mA cm^–2^ at room temperature. A notable drawback is the reliance on a polymer electrolyte layer, which is required for enhanced ionic conductivity for successful charge and discharge.^15^ Luo et al. introduced a novel cobalt/nitrogen-doped carbon material for Na–K alloy electrodes, demonstrating stability with over 1800 cycles at 0.4 mA cm^–2^ and achieving 122.6 mAh g^–1^ capacity at 0.5 C in full cells, the design of which could lower K^+^ diffusion energy barriers and enhance charge transfer. However, the doping process required an argon atmosphere at 800 °C, adding complexity to practical production.^16^ Landmann et al. demonstrated liquid metal anodes’ capabilities by achieving a current density of 2600 mA cm^–2^ at high temperatures of 250 °C without dendrite formation, where cumulative plating capacities exceeding 10 Ah cm^–2^ at 1000 mA cm^–2^ are enabled.^17^ By eliminating creep-related mass transport limitations, they revealed that liquid metal anodes can effectively pair with solid electrolytes. The high-temperature findings hold promise to increase current density by 2 orders of magnitude when translated to room-temperature applications, potentially revolutionizing metal anode design in next-generation batteries.^17^

Despite the promise that liquid alkali metals hold for next-generation batteries, a comprehensive understanding of Na–K alloys’ wetting behavior under battery-relevant conditions is currently missing. As a consequence, liquid alkali metals cannot yet realize their full potential in electrochemical applications. Wetting, defined as the ability of a liquid to displace gas and establish contact with a solid surface, significantly influences battery performance. Parameters such as electrical conductivity, mechanical interfacial contact, and long-term stability are directly impacted by wetting behavior.^13,18−21^ According to Young’s equation, wetting phenomena can be quantified through contact angles.^22^ When a liquid spreads across the surface of the solid, a higher surface tension generally leads to a higher contact angle, which indicates the liquid is more repelled by the solid. Conversely, a lower surface tension results in a lower contact angle, implying better wettability and greater adhesion to the surface. The contact angles reflect the equilibrium of forces at the solid–liquid–gas interface and are inherently governed by the surface tension of the liquid.^23^

Surface tension measurements can be conducted using methods such as the pendant drop method, bubble pressure method, Wilhelmy plate method, and Du Noüy ring method.^24−27^ Since its introduction in 1925, the Du Noüy ring method has been the focus of extensive research due to its high-quality surface tension results.^27−29^ Unlike the pendant drop and bubble pressure methods, which often require complex image analysis or high-pressure systems, the Du Noüy ring method provides a straightforward surface tension analysis based on a force measurement. Moreover, the Du Noüy ring method overcomes the limitations associated with prewetting and surface roughness that can affect the accuracy of the Wilhelmy plate method, making it a versatile and reliable tool for characterizing surface tension in a wide range of liquid systems. Despite these advantages, the Du Noüy ring method has not yet been used to characterize liquid alkali metals. As a consequence, the understanding of the surface tension of alkali metals and their alloys, particularly Na–K alloys, remains incomplete. Existing data on Na–K alloys are especially limited, with most originating between 1950 and 1970. These early studies suffer from unclear methodologies, significant discrepancies, and questionable reliability, leaving a critical gap in our understanding of Na–K surface tension.^30−35^ Differences in experimental conditions, including temperature ranges, measurement techniques, and metal purity, likely contribute to these inconsistencies. Many past studies lacked strict oxygen and moisture control, leading to potential oxidation and contamination, which can significantly alter surface tension measurements. Additionally, improper assumptions, including perfect heat transfer in the maximum bubble pressure method, zero contact angle in the capillary rise method, and reliance on imaging quality in the sessile drop method, introduce further uncertainties in past studies. Therefore, obtaining more accurate and precise surface tension data for Na–K alloys is crucial for enabling the rational design of wetting layers and partially liquid anodes in advanced applications. Such insights will play a key role in optimizing interfaces for improved performance and stability in high-performance batteries.

This work addresses this gap by employing the Du Noüy ring tensiometry method to systematically study the surface tension of Na, K, and Na–K alloys across a range of compositions and temperatures. The findings reveal a clear temperature dependence of surface tension, with values decreasing as temperature increases. Results for Na and K align well with established literature, validating the experimental approach. However, significant deviations are observed when comparing Na–K alloy data to predicted values based on the simplified model from the International Atomic Energy Agency (IAEA), underscoring the importance of direct measurement for accuracy. When evaluating surface tension via the Du Noüy ring technique, the extreme force values measured during the push-in and pull-out processes are employed. The push-in process involves moving the liquid upward to measure surface tension by monitoring the force during interface penetration. The pull-out process involves moving the liquid downward again from the stationary ring to its complete detachment. The superior reproducibility of the push-in method is demonstrated through repeated measurements, which show remarkably low standard deviations compared to the conventional pull-out method, which faces challenges such as early termination due to poor intrinsic wetting. This work further explores the discrepancy between experimental results and the IAEA’s simplified model by employing a thermodynamic model based on the Gibbs free energy minimization method.^36−39^ This model shows excellent agreement with the measured results in this study and accounts for the non-ideal behavior and potassium-rich surface layers in Na–K alloys. Based on our Gibbs free energy minimization approach, the average deviation between measurements and modeling is reduced to 2%, a significant improvement compared to the 25% deviation observed with the IAEA model. The model also successfully predicts surface tension values across an extensive range of compositions (sodium mole fractions from 0 to 1) and temperatures (25 to 200 °C), highlighting its robustness and predictive capability. These results advance the understanding of the fundamental wetting behavior in Na–K systems and bridge the gap between fundamental research and practical battery applications. As a result, this work can serve as a foundation for the design of high-performance, post-lithium-ion batteries, enhancing their durability, scalability, and performance for diverse energy storage applications.

## Experimental Section

### Materials

**Figure 1:**
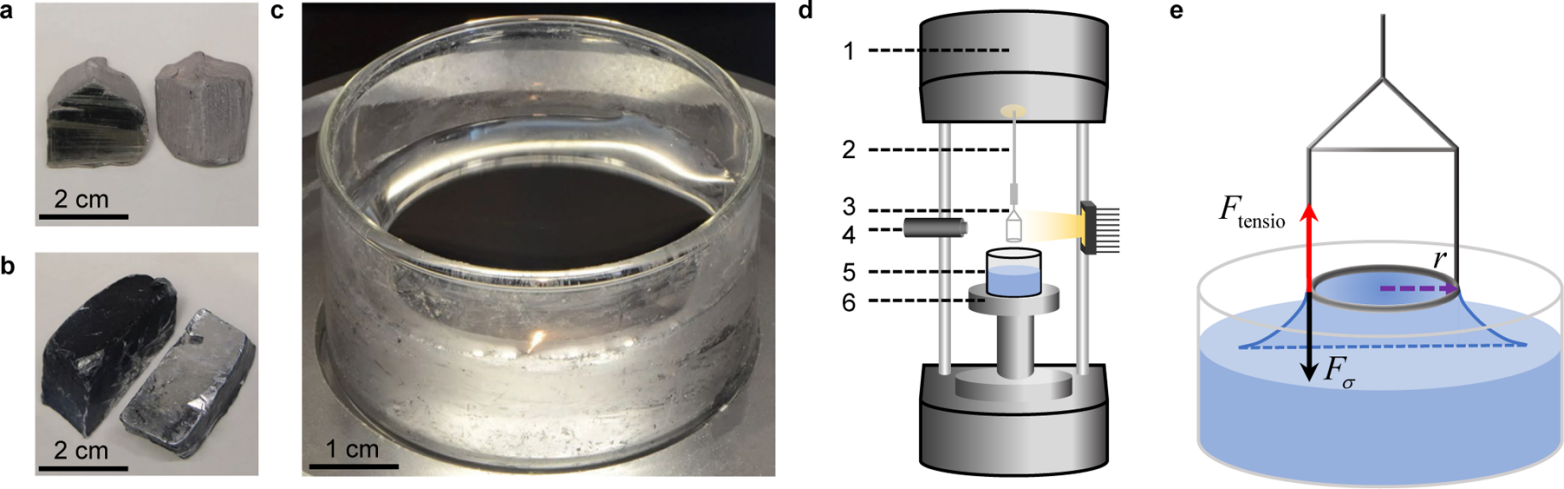
Preparation of the Na–K alloy and introduction of the Du Noüy ring tensiometry method. (a,b) Photographs of fresh-cut sodium and potassium in the glovebox, respectively. (c) Photograph of prepared Na_68_K_32_ alloy in the tensiometer. (d) Schematic of a tensiometer showing a Du Noüy ring (3) attached to an ultrasensitive balance (1) with a clamp (2). The force acting between the liquid and the Du Noüy ring is recorded when a beaker filled with the test liquid (5) is brought in contact with the ring via the vertical movement of a stage (6). The whole process can be captured via a camera (4). (e) Basic working principle of a classical Du Noüy ring measurement, where the surface tension is evaluated from the maximum measured force *F*_tensio_ (equal to the maximum capillary force ) when a Du Noüy ring with an average radius *r* is pulled out from the liquid.

Sodium (purity 99.8%) and potassium (purity 98%) were purchased from Thermo Scientific. Both chemicals were used without further purification (as shown in Figure 1a,b).

### Sample Preparation

To investigate the surface tension of the Na–K system across the full composition range, measurements were conducted on pure sodium, pure potassium, and two Na–K alloys. To this end, two Na–K alloys with different compositions were prepared by mixing sodium and potassium in corresponding weights, namely one alloy with 32.4 mol % (eutectic) and one alloy with 67.5 mol % of sodium. Due to their high reactivity with oxygen and water, all preparations and experiments were carried out in an Ar-filled glovebox under a controlled environment (H_2_O < 0.1 ppm, O_2_ < 0.1 ppm). Before the preparation, a ceramic knife was used to remove the surface contaminants on the alkali-metal sticks. To prepare 30.00 g eutectic Na–K alloy (Na_32_K_68_), 6.60 g of sodium was mixed with 23.40 g of potassium at room temperature. Subsequently, any remaining surface contaminants were carefully removed from the Na–K alloy using a spoon made of stainless steel. The prepared alloy was then ready for surface tension measurements (as shown in Figure 1c). Another composition of Na–K alloy (Na_68_K_32_) was prepared similarly. The pure alkali metals were prepared by adding the required weight of metals in the beaker in the tensiometer at room temperature and heating up to the corresponding melting point to remove the residue surface contaminants.

### Ring Tensiometry Setup

Tensiometry is a key method for measuring surface tension based on a force-time curve, offering ideal reproducibility and high precision. A schematic of the tensiometer setup is illustrated in Figure 1d. The core setup of ring tensiometry features an elaborate platinum–iridium Du Noüy ring (3) attached to an ultrasensitive balance (1) through a clamp (2). The whole setup also includes a stage (6) that moves vertically, enabling precise push-in or pull-out of the ring from the liquid in a beaker (5) at a fixed speed. The entire wetting interaction between the ring and the liquid can be captured by a camera (4) in real time from a side-view perspective. This provides visual feedback and further evidence of the solid–liquid interaction. Benefiting from the ultrasensitive balance, high-precision surface tension measurements with a maximum deviation of 0.01 mg and a sampling rate of 50 Hz is achieved.

### Surface Tension Measurements

The surface tension measurements were performed with a DCAT25 tensiometer manufactured by DataPhysics. The alkali-metal samples were placed into a heating chamber, where the temperature could be adjusted between room temperature and 180 °C. The temperature of the liquid metal was monitored using a built-in thermocouple, while an additional thermocouple measured the chamber atmosphere. All experiments were conducted in an Ar-filled glovebox with a controlled environment.

The procedure for each series of measurements comprised three steps: (1) Outside the glovebox, the Du Noüy ring was flame-treated. Due to the small thermal mass of the thin Du Noüy ring wire, a 5 min waiting period in air is applied for the Du Noüy ring to cool back down to room temperature. (2) The Du Noüy ring was transferred into the glovebox and assembled into the tensiometer at the selected temperature. After a 5 min waiting time, the measurement was started. (3) The measurement was repeated twice. Subsequently, the Du Noüy ring was transferred out of the glovebox to be cleaned again. At least three series of these measurements were performed for each temperature and alloy composition.

### Data Analysis

The raw data output from the tensiometer is the measured weight at the Du Noüy ring probe versus time. By setting a fixed stage speed and accounting for gravitational acceleration, a force–position curve is generated. The principle of classical Du Noüy ring tensiometry, based on the pull-out method, is shown in Figure 1e. Here, the surface tension is determined from the maximum measured force when a circular ring with an average radius, *r*, is pulled out from the liquid. The measured force, *F*_tensio_, is considered as the sum of the capillary force, *F*_σ_, and buoyancy, *F*_b_. In the classical Du Noüy ring method, the maximum measured force above zero is selected for surface tension calculation and the buoyancy is considered as zero. The surface tension, σ, is calculated via eq 1:
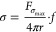
1

Where  is the maximum capillary force, *f* is the correction factor, and *r* is the average radius of the Du Noüy ring, i.e., the mean value between the outer and the inner radius of the ring.^40^ Due to the asymmetric meniscus formed around the ring and the influence of the ring’s dimension, *f* has to be applied in the calculation.^41,42^ In contrast, for the push-in method, the minimum measured value below zero is selected. Using density values from literature and the displaced volume from the immersed ring, the buoyancy component is subtracted from *F*_tensio_. However, there is no reported model for evaluating the correction factor in the push-in method. Aiming at a clearer comparison between these two methods and their data analysis, *f* is set as 1 for both methods in this work. The data points in Figure 3b represent the average values of at least nine measurements, with the error bars indicating the standard deviation.

## Results and Discussion

**Figure 2:**
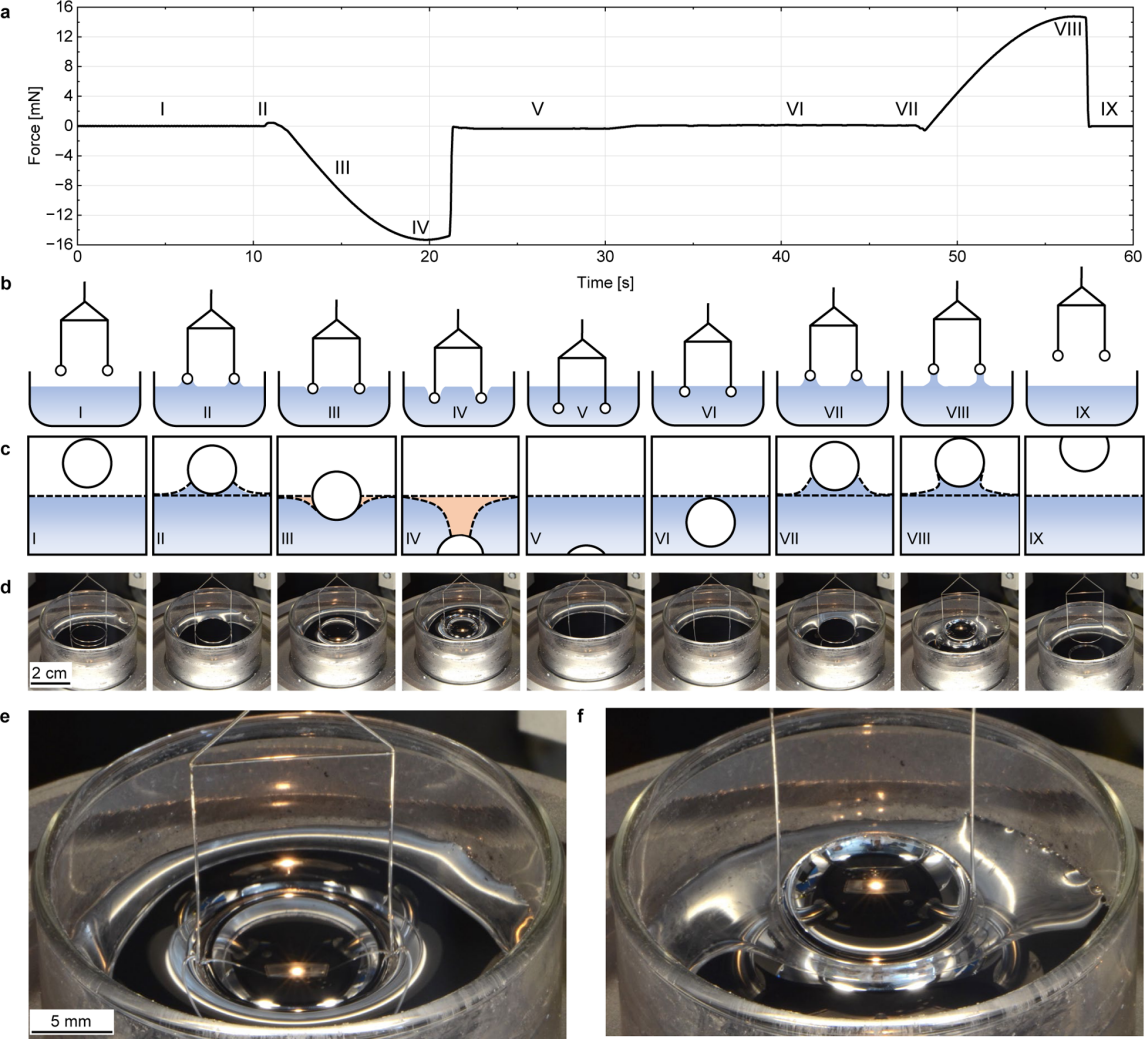
Du Noüy ring measurements of Na–K alloys. (a) Force acting on the Du Noüy ring versus time during a representative measurement with Na_68_K_32_ at 110 °C, also labeling nine important stages of the measurement: (I) Before contact, (II) First contact, (III) Begin of push-in process and meniscus growth, (IV) Continuous growth of the meniscus until the breakpoint, (V–VI) Near force-free motion in liquid metal during push-in and pull-out process, (VII) Second contact between ring and interface, (VIII) Continuous growth of the meniscus until the breakpoint, (IX) End of the measurement. (b) Schematic of the macroscopic interactions between the Du Noüy ring and the liquid surface. (c) Schematic of the microscopic interactions between the Du Noüy ring and the liquid alkali metal. (d) Photographs from an angled perspective showing the interactions between the Du Noüy ring and Na–K eutectic alloy at room temperature. (e) A zoom-in view focused on stage IV during the push-in process. (f) A zoom-in view focused on stage VIII during the pull-out process.

A force-time curve of a representative surface tension measurement on Na_68_K_32_ is shown in Figure 2a, which is obtained via Du Noüy ring tensiometry at 110 °C. In this 60-s measurement, a force change ranging from about −16 mN to 14 mN is recorded. By analyzing the measured force versus time, the whole measurement process can be comprehensively divided into nine stages from the initial approach to the final detachment. It is important to note that during the experiments, the ring remains stationary relative to the balance, while the container holding the liquid alkali metal is moved upward (push-in process) or downward (pull-out process) by the stage. The force measured by the balance reflects the interaction between the ring and the liquid, which changes as the liquid is pushed into or pulled out from the ring. A positive force corresponds to a downward pull exerted by the liquid on the ring, while a negative force corresponds to an upward push. During Stage I, the beaker containing liquid Na–K alloy is slowly lifted toward the Du Noüy ring. Stage II marks the first contact between the ring and the liquid surface, where a slight positive force is recorded during the initial wetting of the Du Noüy ring. As the liquid is moved further upward, immersing the ring in Stage III, a negative force is generated as a result of the surface tension. The measured force reaches a minimum in Stage IV after undergoing a progressive decrease period of up to 9 s from Stage III. It is noteworthy that this prolonged decrease is unique to high-surface-tension fluids like Na_68_K_32_, and is typically not observed in classical application scenarios such as water or silicone oil, as shown in Figure S1. In Stage V, the Du Noüy ring fully overcomes the resistance caused by surface tension and penetrates the liquid–gas interface. The liquid then continues moving upward until a preset immersion depth. As the liquid begins to move downward and the ring is pulled out from the liquid in Stage VI, the measured force shifts to positive, indicating the change of motion direction. Compared to the near-zero force in Stages V and VI, an obvious decrease in force is observed as the liquid level approaches the ring again in Stage VII. By Stage VIII, the registered force steadily increases to its peak, representing the maximum positive capillary force exerted by the liquid on the ring, followed by a decrease just before the actual final detachment in Stage IX. Alongside force changes during measurements, physical interaction between the ring and the liquids also differs, which is revealed by a macroscopic schematic in Figure 2b. A meniscus forms around the ring at the first contact in Stage II and continuously grows downward during the push-in process (Stages III–IV). During the pull-out process, the meniscus reverses its growth direction during Stage VII and continues to rise until detachment in Stage IX. Figure 2c provides a detailed microscopic schematic of the ring’s interaction with the liquid interface. The expanding orange area in Stage III–IV highlights the meniscus propagation during the push-in process, while the deformed meniscus in Stage VIII represents the moment before the final detachment. Serving as visual verification of the schematics and force-time curve, Figure 2d shows nine angled photographs of the ring’s interaction with the eutectic Na–K at room temperature, allowing a direct comparison between theoretical and practical outcomes. For instance, the increased grayish reflected area around the ring in images corresponding to Stages II, VII, and VIII confirms meniscus formation above the liquid surface, as shown in Figure 2b,c. Additionally, a comparison between images for Stages III and IV provides direct evidence of the meniscus growth below the liquid surface during the push-in process. Figure 2e,f provide a zoom-in view focused on stages IV and VIII during the measurement. A video of a Du Noüy ring measurement on Na_68_K_32_ at room temperature is provided in the Supporting Information for further reference (Video S1).

**Figure 3:**
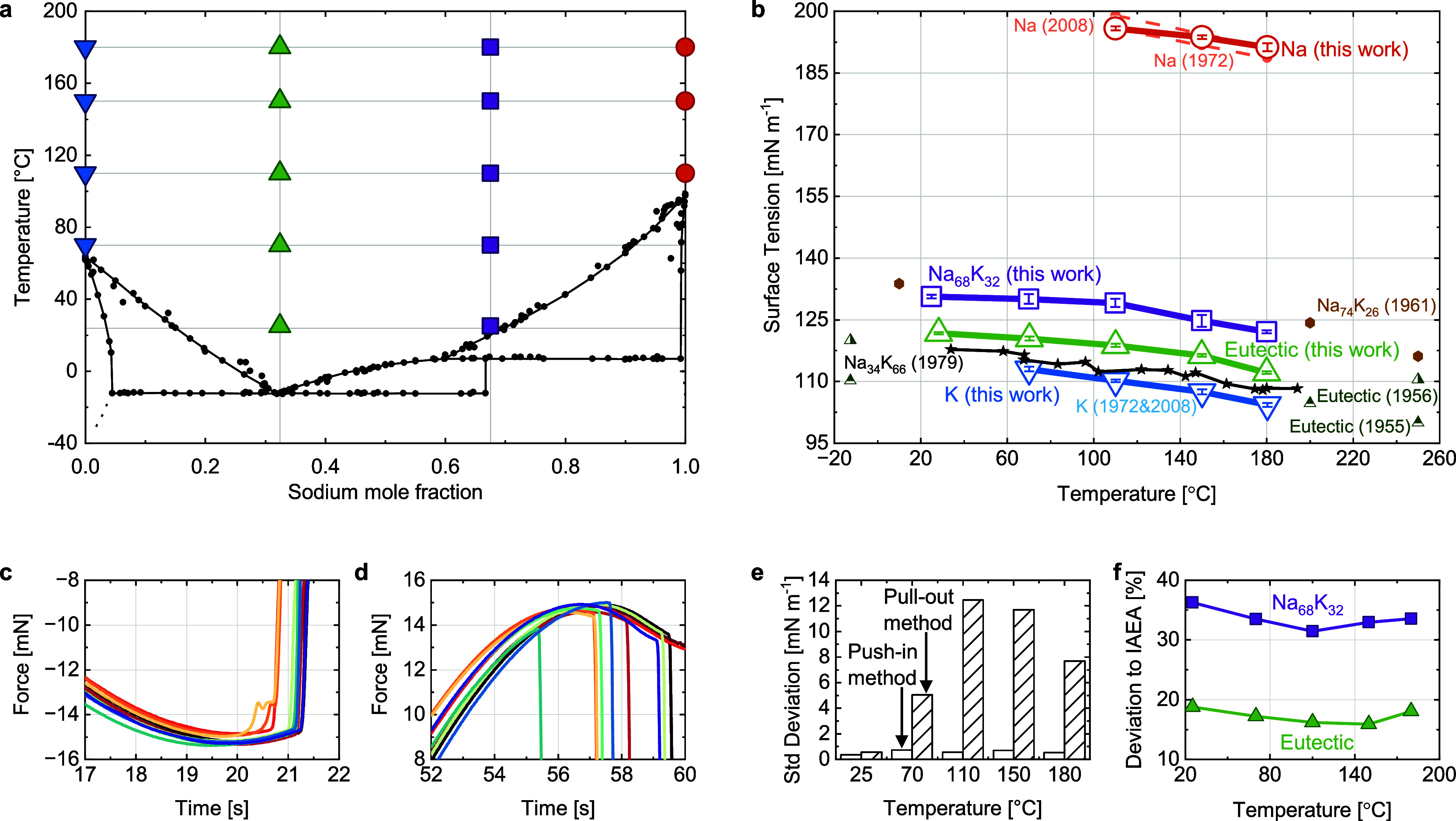
Experimental plan and results of surface tension measurements. (a) Na–K phase diagram introducing the experimental plan. The colored symbols show the Na–K compositions and temperatures at which the measurements were performed. (b) Surface tension of pure K, pure Na, and selected Na–K alloys, with 32 mol % and 68 mol % sodium, respectively, as a function of temperature. Each data point represents the average of at least eight individual measurements and the error bar indicates the corresponding standard deviation. (c) Comparison of the minimum force in raw data from 11 measurements with Na_68_K_32_ at 110 °C. (d) Comparison of the maximum force in raw data from 11 measurements with Na_68_K_32_ at 110 °C. (e) Standard deviations during push-in and pull-out methods averaged over measurements across all temperatures studied in this work. (f) Deviation in percent between the measurements performed in this work as compared to a simplified IAEA model in the literature.^31^ The deviation is defined by (σ_predicted_–σ_measured_)/σ_measured_ × 100%.

The Na–K phase diagram is plotted in Figure 3a based on literature data.^43−47^ The diagram shows a wide liquid-phase region between 15 mol % and 70 mol % sodium at room temperature with the melting point of the eutectic alloy (32.4 mol % sodium) as low as −12.6 °C. The experimental plan followed in the present study, mapped with colored symbols in the phase diagram, spans the compositional and temperature ranges of experimental interest (ambient, 70 °C, 110 °C, 150 °C, and 180 °C), including Na, K, Na_68_K_32_ and eutectic Na–K alloy. During the initial experiments on Na_68_K_32_, significant limitations of the traditional pull-out method became evident. This can be attributed to the poor intrinsic wetting between the Du Noüy ring and the liquid Na–K alloys. Minor perturbations in the experiments can then result in a premature detachment between the ring and the liquid alloy, which in turn leads to a pronounced deviation between individual measurements. In contrast, the push-in method showed substantially lower deviations between individual measurements, thereby drastically enhancing the quality of the measurements. The detailed data for Na_68_K_32_ measurements from ambient to 180 °C is plotted in Figures S2–S6.

The results evaluated via the push-in method along with selected literature values are presented in Figure 3b. A clear trend of decreasing surface tension with increasing temperature is demonstrated, which is consistent with expectations. For pure sodium and potassium, the measured values align closely with the recommended temperature-dependent equations from the literature, with differences as small as 3 mN m^–1^ and an overall deviation as low as 0.3%, defined by (σ_literature_ – σ_measured_)/σ_measured_ × 100%, validating both the experimental approach and the accuracy of the push-in method.^30,31^ In contrast, surface tension data for Na–K alloys are less explored and existing literature is often inconsistent. The existing surface tension values from the literature for the Na–K system are summarized in Figure S7.^30−35^ Certain data were excluded in this summary due to large errors or poor readability, including an unexpected value lower than that of pure potassium and an unrealistically low value reported at 100 °C.^48,49^ From the full summary in Figure S7, selected high-quality data is also included in Figure 3b for comparison. Our measurements show, that the surface tension of Na_68_K_32_ decreases from 130.6 mN m^–1^ to 122.1 mN m^–1^ as the temperature increases from ambient to 180 °C, while the surface tension of the eutectic alloy decreases from 121.7 mN m^–1^ to 112.2 mN m^–1^ over the same temperature range. These findings for the eutectic alloy are consistent with near-eutectic data (Na_34_K_66_) reported by Petrick et al. via the maximum bubble pressure method in 1979.^34^ Similarly, results from Sittig et al. align closely with this work. For Na_68_K_32_, the measured values are validated by reported sessile-drop-method data for Na_74_K_26_, which, due to its slightly lower potassium content, has slightly higher surface tension. Detailed values are listed in Table S1.

The precision and reproducibility of the Du Noüy ring push-in method proposed here are illustrated in Figure 3c, which showcases the minimum force recorded during 11 repeated measurements of Na_68_K_32_ at 110 °C. Compared to the corresponding maximum values during the pull-out moment shown in Figure 3d, the measurements with the push-in method generally demonstrate remarkable consistency with a standard deviation as low as 1 mN m^–1^. Figure 3e provides a direct comparison of the push-in and pull-out methods by analyzing the average standard deviations of all the measurements in this study across various temperatures. At ambient temperature, the difference between the push-in method and the pull-out method is relatively small. At higher temperatures such as 110 °C, however, the average standard deviation drastically increased from 0.6 mN m^–1^ for the push-in method to 12.5 mN m^–1^ for the pull-out method. This trend is similarly observed at other temperatures, underscoring the superiority of the push-in method for systems with high surface energy such as Na–K alloys. Averaged standard deviation data are detailed in Table S2, while the standard deviations of push-in and pull-out methods for the four individual compositions studied in our work are shown in Figure S8. The results for Na–K alloys exhibit significant discrepancies when compared to the values predicted by the IAEA using the simplified model.^31^Figure 3f highlights the deviation in percent between the measured values as compared to the predicted values from the IAEA. For Na_68_K_32_, a deviation of up to 35% was observed, while the eutectic alloy showed a deviation of approximately 20%. Compared to predicted values, experimental results obtained in this study are more reliable, as corroborated by their consistency with other experimentally derived data.

The significant discrepancy between experimental results and the simplified model recommended by the IAEA highlights the need to explore its theoretical foundation. The IAEA’s model is based on the ideal behavior of solutions, as described by Raoult’s law, where the surface tension of the solution is assumed to be the mole-weighted average of the individual surface tensions. However, both theoretical and experimental studies have demonstrated that non-ideal behaviors, such as activity coefficients and excess energy, must also be taken into account. Since 1932, researchers have developed and validated a reasonable thermodynamic model to predict surface tension using the Gibbs free energy minimization method.^36−39^ For instance, Tanaka et al. applied this approach to six binary systems at 873 K, demonstrating excellent agreement with available experimental data.^38^ Pajarre et al. also implemented the model for the binary Bi–Sn system at 608 K, reporting a deviation of up to 10 mN m^–1^.^37^ This result inspires the first-time application of this model to the Na–K system explored in this study. In the Gibbs free energy minimization method, the total Gibbs free energy (*G*^total^) of a binary system combines contributions from the bulk phase (*G*^bulk^) and the surface phase (*G*^surface^), with the surface treated as a monolayer of atoms. The Gibbs free energy in each phase is calculated as eqs 2 and 3.
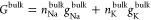
2
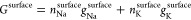
3

At equilibrium, the total Gibbs free energy *G*^total^ is minimized, ensuring chemical potential uniformity for each component across phases. For non-ideal binary systems, the surface phase introduces additional complexity due to interfacial anisotropy, which requires modifications on the Gibbs free energy of each phase. The partial Gibbs free energy of sodium in the bulk phase can be expressed as shown in eq 4, where  is the molar Gibbs free energy of pure sodium in the bulk phase, representing the ideal behavior of sodium. The latter two terms account for the non-ideal behavior by incorporating the effects of composition and temperature. Similarly, the partial Gibbs free energy of sodium in the surface phase can be expressed as eq 5,
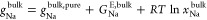
4
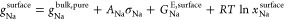
5where the molar Gibbs free energy of pure sodium in the surface phase  consists of an intrinsic contribution from  and an additional term reflecting the effects of sodium’s surface tension (σ_Na_) and molar surface area (*A*_Na_). Moreover, the surface tension of the binary Na–K system contributes to the deviation of the molar Gibbs free energy of sodium in the surface phase from that in the bulk phase by *σA*_Na_, providing a critical link between eq 4 and 5, as shown in eq 6.
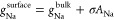
6

The surface tension of Na–K alloys at a given sodium mole fraction () and temperature (*T*) therefore can be expressed as a function of the mole fraction at the surface phase (), as shown in eqs 7 and 8, where σ_*i*_ is the surface tension of pure component *i* at a given temperature *T* and *R* is the ideal gas constant. The excess Gibbs free energy () in both phases is evaluated using the models and equations summarized by Bale and Tanaka et al.^38,47^ The detailed calculation of molar surface area and the full derivation of the model can be found in the Supporting Information.
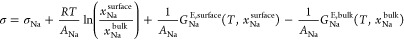
7
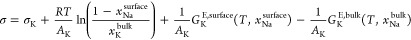
8

**Figure 4:**
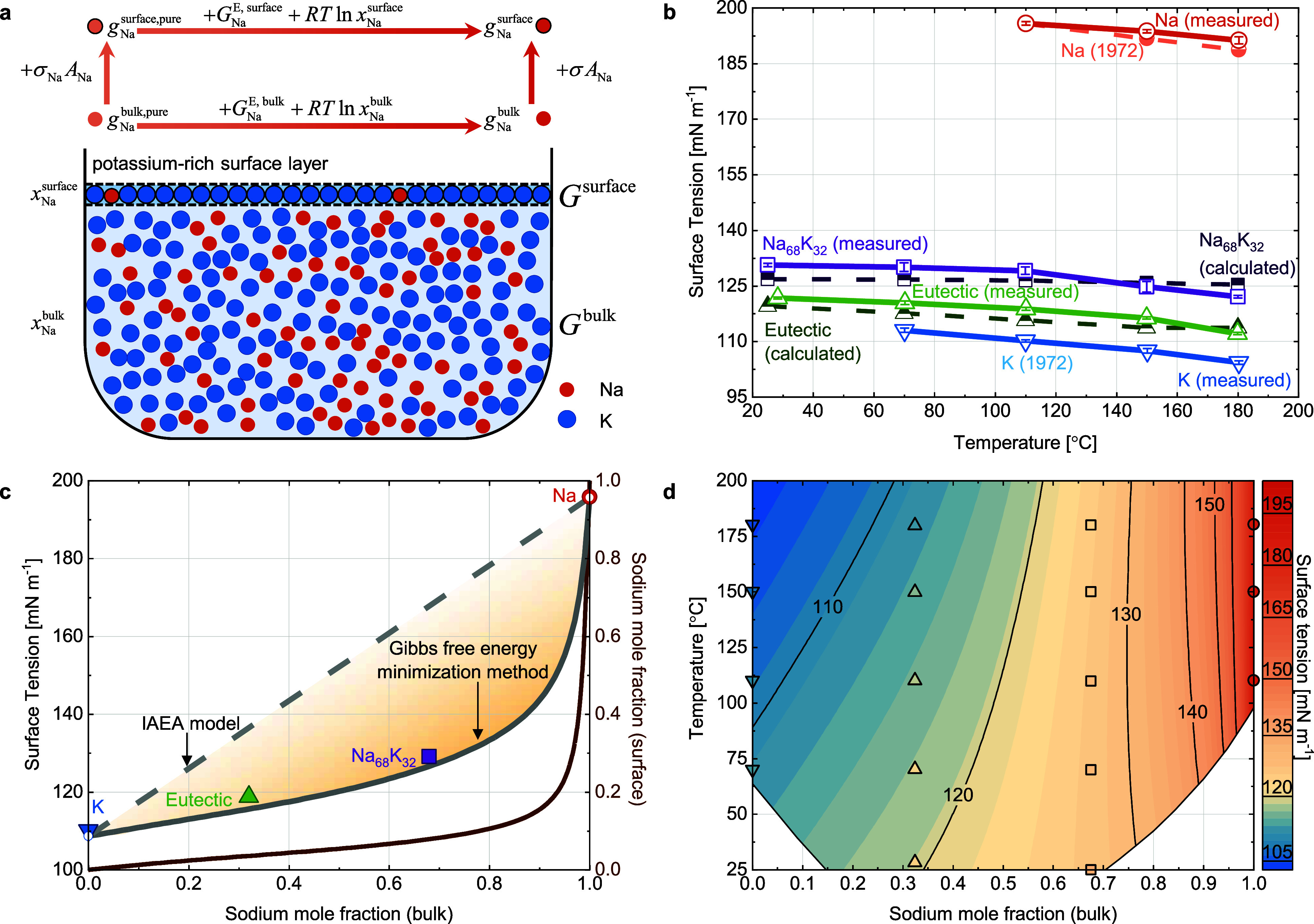
Modeling Na–K surface tension via the Gibbs free energy minimization method. (a) Schematic of the Gibbs free energy minimization method, introducing key variables and their relationships at the example of Na, alongside a schematic of a beaker containing eutectic Na–K alloy. Blue circles represent K atoms, and orange circles represent Na atoms, illustrating the non-ideal behavior of the alloy with a potassium-rich surface layer and a noticeable sodium concentration difference between the surface and bulk phases. (b) Modeled (dashed lines) and measured (solid lines) surface tension of two Na–K alloys across temperatures, compared with literature values for pure components.^30^ (c) Computed surface tension and sodium mole fraction in the surface phase versus sodium mole fraction in the bulk phase at 110 °C, showing a potassium-rich surface layer. Larger colored symbols represent experimental measurements, while hollow symbols indicate literature values. (d) Contour plot computed via the Gibbs free energy minimization method, illustrating the variation of surface tension of the Na–K alloy as a function of sodium mole fraction in the bulk phase and temperature.

To better explain this model, a schematic is presented in Figure 4a, highlighting the non-ideal behavior of the eutectic Na–K alloy. The upper section in Figure 4a illustrates the theoretical thermodynamic relationships within the model, depicting the connection among , , , and . The lower section provides a schematic representation of the eutectic Na–K alloy, which consists of a bulk phase and a surface phase. In this illustration, blue symbols represent K atoms, while orange symbols represent Na atoms. It demonstrates that the is significantly lower than , indicating the existence of a K-rich surface layer. Details about this enrichment will be provided in the next paragraph. Figure 4b compares the modeled and measured surface tension of Na–K alloys, where the solid lines represent experimental data, while dashed lines denote summarized values for pure components from 1972 and the modeled Na–K alloy data.^30^ The model aligns closely with measurements, with a maximum difference of 3.8 mN m^–1^, which leads to a largest deviation of 2.9% (defined by (σ_calculated_ – σ_measured_)/σ_measured_ × 100%). For instance, the modeled surface tension for Na_68_K_32_ at 150 °C is 125.9 mN m^–1^, compared to a measured value of 124.8 mN m^–1^. By contrast, the IAEA’s simplified model shows an average deviation of 25%, highlighting the superior accuracy of the present model, which achieves a reduction in deviation by over an order of magnitude to 2%. Additionally, the extrapolated surface tension of the pure components below their melting points enables accurate predictions for Na–K alloys at ambient and 70 °C, reinforcing both the robustness of the model and the reliability of the experimental measurements (detailed comparisons in Table S3).

Bradhurst et al. observed similar non-ideal behavior and the potassium-rich surface layer in the Na–K system by plotting the non-linear isothermal curves between potassium weight percent and surface tension at the melting point, 200 and 250 °C.^35^ This finding is corroborated by the present model, as illustrated in Figure 4c, which presents an isothermal curve between the mole fraction of sodium in the bulk phase and the surface tension of Na–K alloys at 110 °C, along with the corresponding mole fraction in the surface phase. The non-linear rise in surface tension with increasing sodium mole fraction in the bulk phase underscores the necessity of including activity coefficients or excess Gibbs free energy in the model and explains why the simplified model proposed by the IAEA is not proper for the Na–K system. Moreover, the larger colored symbols for the measured values align closely with the gray line for modeled values and the hollow symbols for pure components’ surface tension, further confirming the accuracy of both the model and the experimental measurements. The delayed increase in sodium mole fraction in the surface phase relative to the bulk phase confirms this potassium enrichment. For example, at a bulk sodium mole fraction of 0.90, the surface mole fraction is only 0.15. Detailed results are provided in Table S4. Zhang et al. have also found this potassium-rich surface layer in eutectic Na–K alloy and utilized it as a protective barrier between the Na–K alloy and the electrolyte while improving wettability between the liquid Na–K alloy and a carbon matrix.^50^ Building on the successful predictions for the two Na–K alloys in this study, the model was extended to a broader range of compositions (sodium mole fractions in the bulk phase ranging from 0 to 1 in 0.01 increments) and temperatures (25 to 200 °C in 5 °C steps). The modeled surface tension and the corresponding sodium mole fraction in the surface phase are presented in Figures 4d and S9 as contour plots, providing a comprehensive visualization across the full composition and temperature range. The data points in the contour plot represent measured values and the filling colors correspond to the surface tension values, as indicated by the color bar, providing a visual comparison between the modeled and measured results. From the contour plot, it can be generally concluded that the surface tension of Na–K alloys decreases with increasing temperatures, a trend that can be attributed to enhanced molecular movement and reduced intermolecular attraction at higher temperatures. Additionally, the surface tension of Na–K alloys rises as the sodium mole fraction in the bulk phase increases, which is linked to the corresponding increase in the sodium mole fraction in the surface phase. For instance, the sodium’s surface composition is 3 mol % for a bulk composition of 30 mol % and it jumps to 23 mol % for 95 mol %. However, despite this increase, the surface monolayer remains potassium-rich. At a bulk sodium composition of 90 mol %, the surface composition is 8 mol % at 50 °C and remains as low as 30 mol % at 200 °C, as shown in Figure S8. Detailed modeling results are listed in Tables S5 and S6. In summary, the contour plot shown in Figure 4d allows for the first time to obtain high-quality surface tension data for the Na–K system across the full range of composition and up to sufficiently high temperatures for battery applications.

With the precise push-in Du Noüy ring tensiometry and a robust thermodynamic model, a comprehensive understanding of the surface tension of sodium, potassium, and their alloys has been achieved. The surface tension data obtained in this study is valuable for further application in Wilhelmy plate tensiometry, enabling the measurement of dynamic contact angles between alkali metals and other battery materials such as solid electrolytes. These measurements accurately represent the real wetting behavior under practical conditions. Additionally, dynamic contact angle results can be directly derived from the force curve in the Wilhelmy plate tensiometry, overcoming the limitations of conventional static contact angle measurements, which rely on high-resolution cameras, image analysis, and are constrained by surface homogeneity and droplet size. In battery applications, precise wetting control is critical: some components require perfect wetting to reduce the battery cell volume, while others benefit from poor wetting to enhance safety by preventing alkali-metal leakage.^51^ Most importantly, being able to accurately characterize the surface tension of alkali metals enables rationally engineered functional coatings on solid electrolytes for enhanced alkali-metal wetting. Such surface modifications can substantially enhance the contact between the solid electrolyte and the alkali-metal electrode, thereby improving the interfacial charge transfer, heat transfer, and uniformity of electrochemical reactions.^52,53^ Moreover, surface tension directly affects capillary phenomena in Na–K alloys, which can support the design of reservoir-free alkali-metal batteries.^54^ It is also reported that the surface tension of Na–K alloys can be engineered via additives. For instance, adding 0.15 wt % of barium to a eutectic Na–K alloy can reduce the surface tension by approximately 8%. However, after several attempts, challenges were encountered with dissolving barium into the Na–K system, likely due to different purity and dissolving techniques. We also explored adding Sn to the Na–K system to lower surface tension, observing a reduction in surface tension of around 4% when adding 22.5 wt % of Sn powder. These preliminary findings suggest that there is room for further engineering surface tension of Na–K alloys. Additionally, the innovative push-in Du Noüy ring tensiometry opens up broader applications for high-surface-tension liquids, including liquid electrolytes and other potential liquid alloys, thus expanding the scope for next-generation batteries.

## Conclusion

In this study, we explored the surface tension of Na–K alloys, a critical property for understanding wetting phenomena in alkali-metal battery applications. Using precise Du Noüy ring tensiometry, we investigated the surface tension behavior of Na–K alloys across various temperatures and compositions. Our findings revealed that surface tension decreases with increasing temperature, consistent with thermodynamic principles, and increases with higher sodium mole fractions in the bulk phase. However, the surface remains predominantly potassium-rich, indicating non-ideal surface behavior, which was confirmed through computation based on Gibbs free energy minimization. The proposed push-in method for Du Noüy ring tensiometry provided significantly better precision and consistency than the traditional pull-out method, with an average standard deviation of 1 mN m^–1^ compared to over 12 mN m^–1^ in the pull-out method at 110 °C. This method allowed for more reliable measurements, particularly for Na–K alloys, where poor intrinsic wetting often causes early detachment in pull-out tests. Our experimental results represent the first high-quality, systematic surface tension data for Na–K alloys across a broad temperature range and various compositions. These findings were validated against literature values, confirming the reliability of our measurements and underscoring the superiority of our approach. Moreover, the study highlighted the limitations of the IAEA’s additivity model, demonstrating the need for a more accurate predictive framework. To this end, we introduced a model, incorporating both ideal and non-ideal contributions, including excess Gibbs free energy and activity coefficients, to successfully predict surface tension with minimal deviation from experimental data. In conclusion, this study not only provides valuable insights into the wetting behavior of Na–K alloys but also establishes an innovative experimental approach and a robust predictive model based on Gibbs free energy minimization. These results fill a critical gap in the understanding of Na–K alloy wetting behavior and offer valuable data for applications in energy storage and beyond. This comprehensive work highlights the interplay between experimental innovation and advanced modeling, setting a new benchmark for studying the wetting behaviors of alkali-metal alloys.

## Supporting Information Available

Detailed derivation about the surface tension calculation model, Du Noüy ring measurements of silicone oil, raw data for Na_68_K_32_ measurements at room temperature, 70 °C, 110 °C, 150 °C, and 180 °C, comparison between existing values in literature and measured values in this work for the Na–K system, contour plot about sodium mole fraction in the surface phase of the Na–K alloy as a function of sodium mole fraction in the bulk phase and temperature, detailed data about measured values, and standard deviations in table form, and calculated surface tension and sodium mole fraction in the surface phase in table form (PDF)
A representative video of a Du Noüy ring measurement on Na_68_K_32_ at room temperature (MP4)
