# Supplementary material for: High-Precision Surface Tension Measurements of Sodium, Potassium, and Their Alloys via Du Noüy Ring Tensiometry
Source: ACS Appl Mater Interfaces. 2025 Apr 21;17(17):25985–95. doi: 10.1021/acsami.5c02183 (PMC12051173; doi:10.1021/acsami.5c02183)
Supplement: Supplementary file 1 — am5c02183_si_001.pdf [file am5c02183_si_001.pdf]

Supporting Information

High-precision Surface Tension Measurements of Sodium, Potassium, and Their Alloys via Du Noüy Ring Tensiometry

Naiyu Qi[1], Rachana Somaskandan[1,2] and Gustav Graeber[1,3,+]

[+]gustav.graeber@hu-berlin.de

[1]Graeber Lab for Energy Research, Department of Chemistry, Humboldt-Universität zu Berlin, 12489 Berlin, Germany

[2]Department of Chemical Engineering, Northeastern University, Boston, MA 02115, USA

[3]Department 3: Containment Systems for Dangerous Goods; Energy Storage, Federal Institute for Materials Research and Testing, (BAM), 12205 Berlin, Germany

Detailed derivation about the surface tension calculation model

This model is partially introduced by Kang et al. and here we develop it for Na-K application.[1]

Following Butler et al., the total Gibbs free energy (G^{total}) of a binary system is expressed as the sum of contributions from the bulk (G^{bulk}) and surface (G^{surface}) phases, with the surface phase approximated as a monolayer of atoms.[2] The total Gibbs free energy can be written as:

$$G^{\text{total}} = G^{\text{bulk}} + G^{\text{surface}} \tag{1}$$

If the Gibbs free energy of the bulk phase is represented relative to the corresponding number of moles of a component i in each phase ($n_i^{\text{bulk/surface}}$), the partial Gibbs free energy of a component i in the bulk phase (g_i^{bulk}), the surface tension (σ), and the surface area (A), **Equation (1)** can be rewritten as:

$$G^{\text{total}} = (n_{\text{Na}}^{\text{bulk}} g_{\text{Na}}^{\text{bulk}} + n_{\text{K}}^{\text{bulk}} g_{\text{K}}^{\text{bulk}}) + (n_{\text{Na}}^{\text{surface}} g_{\text{Na}}^{\text{bulk}} + n_{\text{K}}^{\text{surface}} g_{\text{K}}^{\text{bulk}} + \sigma A) \tag{2}$$

When the surface area of the system (A) is expressed using the molar surface area of a component i as shown in **Equation (3)**, **Equation (2)** can be rearranged as **Equation (4)**:

$$A = n_{\text{Na}}^{\text{surface}} A_{\text{Na}} + n_{\text{K}}^{\text{surface}} A_{\text{K}} \tag{3}$$

$$G^{\text{total}} = (n_{\text{Na}}^{\text{bulk}} g_{\text{Na}}^{\text{bulk}} + n_{\text{K}}^{\text{bulk}} g_{\text{K}}^{\text{bulk}}) + n_{\text{Na}}^{\text{surface}} (g_{\text{Na}}^{\text{bulk}} + \sigma A_{\text{Na}}) + n_{\text{K}}^{\text{surface}} (g_{\text{K}}^{\text{bulk}} + \sigma A_{\text{K}}) \tag{4}$$

In comparison to **Equation (1)**, the term $g_{\text{Na}}^{\text{bulk}} + \sigma A_{\text{Na}}$ can be interpreted as the partial Gibbs free energy of sodium in the surface phase ($g_{\text{Na}}^{\text{surface}}$), which reflects how $g_{\text{Na}}^{\text{surface}}$ is shifted from its value in the bulk phase ($g_{\text{Na}}^{\text{bulk}}$) by σA_{Na}. Considering non-ideal interactions in a binary system, the activity of sodium in the surface phase is introduced as $a_{\text{Na}}^{\text{surface}}$. As shown in **Equation (5)**, $g_{\text{Na}}^{\text{surface}}$ can also be expressed as the sum of the molar Gibbs free energy of pure sodium in the surface phase ($g_{\text{Na}}^{\text{surface,pure}}$) and $RT \ln a_{\text{Na}}^{\text{surface}}$, where the R is the ideal gas constant and T is the corresponding temperature. For simplification, the following derivation will focus on sodium.

$$g_{\text{Na}}^{\text{surface}} \equiv g_{\text{Na}}^{\text{bulk}} + A_{\text{Na}} \sigma = g_{\text{Na}}^{\text{surface,pure}} + RT \ln a_{\text{Na}}^{\text{surface}} \tag{5}$$

From **Equation (5)**, various expressions for $g_{\text{Na}}^{\text{bulk}}$ can be derived and are provided as **Equations (6) – (8)**:

$$g_{\text{Na}}^{\text{bulk}} = g_{\text{Na}}^{\text{bulk,pure}} + RT \ln a_{\text{Na}}^{\text{bulk}} \tag{6}$$

$$g_{\text{Na}}^{\text{bulk}} = g_{\text{Na}}^{\text{surface}} - A_{\text{Na}} \sigma \tag{7}$$

$$g_{\mathrm{Na}}^{\mathrm{bulk}} = g_{\mathrm{Na}}^{\mathrm{surface,pure}} + RT\ln a_{\mathrm{Na}}^{\mathrm{surface}} - A_{\mathrm{Na}}\sigma \quad (8)$$

For a system consisting solely of sodium, the following relations can be established, allowing $g_{\mathrm{Na}}^{\mathrm{surface,pure}}$ to be written in **Equation (10)**:

$$a_{\mathrm{Na}} = a_{\mathrm{Na}}^{\mathrm{surface}} = 1, A_{\mathrm{Na}} = A_{\mathrm{Na}}^{\mathrm{pure}}, \sigma = \sigma_{\mathrm{Na}} \quad (9)$$

$$g_{\mathrm{Na}}^{\mathrm{surface,pure}} = g_{\mathrm{Na}}^{\mathrm{bulk,pure}} + A_{\mathrm{Na}}^{\mathrm{pure}}\sigma_{\mathrm{Na}} \quad (10)$$

Substituting **Equation (10)** into **Equation (8)**, $g_{\mathrm{Na}}^{\mathrm{bulk}}$ can be expressed as:

$$g_{\mathrm{Na}}^{\mathrm{bulk}} = g_{\mathrm{Na}}^{\mathrm{bulk,pure}} + A_{\mathrm{Na}}^{\mathrm{pure}}\sigma_{\mathrm{Na}} + RT\ln a_{\mathrm{Na}}^{\mathrm{surface}} - A_{\mathrm{Na}}\sigma \quad (11)$$

Combining **Equation (11)** and **Equation (6)**, the surface tension of the mixture (σ) can be expressed as a function of temperature and activity of sodium, as shown in **Equation (12)**. The activity coefficient (γ_{Na}) is introduced to quantify deviations from ideal behavior, where the activity of sodium (a_{Na}) is written as $a_{\mathrm{Na}} = \gamma_{\mathrm{Na}} * x_{\mathrm{Na}}$, where the x_{Na} means the molar fraction of sodium in each phase.

$$\begin{aligned}
\sigma &= \sigma_{\mathrm{Na}}\frac{A_{\mathrm{Na}}^{\mathrm{pure}}}{A_{\mathrm{Na}}} + \frac{RT}{A_{\mathrm{Na}}}\ln\left(\frac{a_{\mathrm{Na}}^{\mathrm{surface}}}{a_{\mathrm{Na}}^{\mathrm{bulk}}}\right) \\
&= \sigma_{\mathrm{Na}} + \frac{RT}{A_{\mathrm{Na}}}\ln\left(\frac{a_{\mathrm{Na}}^{\mathrm{surface}}}{a_{\mathrm{Na}}^{\mathrm{bulk}}}\right) \\
&= \sigma_{\mathrm{Na}} + \frac{RT}{A_{\mathrm{Na}}}\ln\left(\frac{x_{\mathrm{Na}}^{\mathrm{surface}}\gamma_{\mathrm{Na}}^{\mathrm{surface}}}{x_{\mathrm{Na}}^{\mathrm{bulk}}\gamma_{\mathrm{Na}}^{\mathrm{bulk}}}\right) \\
&= \sigma_{\mathrm{Na}} + \frac{RT}{A_{\mathrm{Na}}}\ln\left(\frac{x_{\mathrm{Na}}^{\mathrm{surface}}}{x_{\mathrm{Na}}^{\mathrm{bulk}}}\right) + \frac{RT}{A_{\mathrm{Na}}}\ln\left(\gamma_{\mathrm{Na}}^{\mathrm{surface}}\right) - \frac{RT}{A_{\mathrm{Na}}}\ln\left(\gamma_{\mathrm{Na}}^{\mathrm{bulk}}\right)
\end{aligned} \quad (12)$$

Furthermore, the term ($RT\ln(\gamma_{\mathrm{Na}})$) can be simplified to represent the excess Gibbs free energy ($G_{\mathrm{Na}}^{\mathrm{E}}$) in each phase, streamlining the incorporation of non-ideal interactions into the thermodynamic framework. Finally, at a given molar fraction of sodium in the bulk phase ($x_{\mathrm{Na}}^{\mathrm{bulk}}$), the surface tension of the mixture can be calculated as a function of T and $x_{\mathrm{Na}}^{\mathrm{surface}}$. The framework can be extended to potassium by substituting sodium with potassium, allowing the calculation of surface tension using **Equations (13) and (14)**.

$$\sigma = \sigma_{\mathrm{Na}} + \frac{RT}{A_{\mathrm{Na}}}\ln\left(\frac{x_{\mathrm{Na}}^{\mathrm{surface}}}{x_{\mathrm{Na}}^{\mathrm{bulk}}}\right) + \frac{1}{A_{\mathrm{Na}}}G_{\mathrm{Na}}^{\mathrm{E,surface}}(T, x_{\mathrm{Na}}^{\mathrm{surface}}) - \frac{1}{A_{\mathrm{Na}}}G_{\mathrm{Na}}^{\mathrm{E,bulk}}(T, x_{\mathrm{Na}}^{\mathrm{bulk}}) \quad (13)$$

$$\sigma = \sigma_{\mathrm{K}} + \frac{RT}{A_{\mathrm{K}}}\ln\left(\frac{1 - x_{\mathrm{Na}}^{\mathrm{surface}}}{x_{\mathrm{K}}^{\mathrm{bulk}}}\right) + \frac{1}{A_{\mathrm{K}}}G_{\mathrm{K}}^{\mathrm{E,surface}}(T, x_{\mathrm{Na}}^{\mathrm{surface}}) - \frac{1}{A_{\mathrm{K}}}G_{\mathrm{K}}^{\mathrm{E,bulk}}(T, x_{\mathrm{Na}}^{\mathrm{bulk}}) \quad (14)$$

The molar surface area of each component can be computed from the Avogadro constant (N_0) and corresponding molar volume ($V_{\text{Na,K}}$) as shown in **Equation (15)**. For liquid metals, a correction factor (β) of 0.83 must be applied when transitioning from the excess Gibbs free energy of the bulk phase to that of the surface phase.[3] This involves substituting the bulk-phase molar fraction of each component with the corresponding surface-phase molar fraction, as shown in **Equation (16)**.

$$A_{\text{Na,K}} = 1.091 \cdot N_0^{1/3} V_{\text{Na,K}}^{2/3} \quad (15)$$

$$G_{\text{Na,K}}^{\text{E,surface}}(T, x_{\text{Na}}^{\text{surface}}) = \beta \cdot G_{\text{Na,K}}^{\text{E,bulk}}(T, x_{\text{Na}}^{\text{surface}}) \quad (16)$$

The expression for the excess Gibbs free energy in terms of each component is taken from Bale et al. and shown in **Equations (17) and (18)**.[4]

$$G_{\text{Na}}^{\text{E}} = (1 - x_{\text{Na}})^2 ((2251.0 + 2811.6 x_{\text{Na}}) - T(-0.6309 + 2.6007 x_{\text{Na}} + 15.2872 x_{\text{Na}}{}^2 - 23.4092 x_{\text{Na}}{}^3)) \quad (17)$$

$$G_{\text{K}}^{\text{E}} = x_{\text{Na}}{}^2 (845.2 + 2811.6 x_{\text{Na}}) - T x_{\text{Na}}{}^2 (-1.9313 - 7.5908 x_{\text{Na}} + 32.8441 x_{\text{Na}}{}^2 - 23.4092 x_{\text{Na}}{}^3) \quad (18)$$

Deriving from the above equations, G^{bulk} and G^{surface} can be written in **Equations (19) and (20)**.

$$\begin{aligned} G^{\text{bulk}} &= n_{\text{Na}}^{\text{bulk}} g_{\text{Na}}^{\text{bulk}} + n_{\text{K}}^{\text{bulk}} g_{\text{K}}^{\text{bulk}} \\ &= n_{\text{Na}}^{\text{bulk}} (g_{\text{Na}}^{\text{bulk,pure}} + RT \ln x_{\text{Na}}^{\text{bulk}} + G_{\text{Na}}^{\text{E,bulk}}(T, x_{\text{Na}}^{\text{bulk}})) + n_{\text{K}}^{\text{bulk}} (g_{\text{K}}^{\text{bulk,pure}} + RT \ln x_{\text{K}}^{\text{bulk}} + G_{\text{K}}^{\text{E,bulk}}(T, x_{\text{Na}}^{\text{bulk}})) \end{aligned} \quad (19)$$

$$\begin{aligned} G^{\text{surface}} &= n_{\text{Na}}^{\text{surface}} g_{\text{Na}}^{\text{bulk}} + n_{\text{K}}^{\text{surface}} g_{\text{K}}^{\text{bulk}} + \sigma A \\ &= n_{\text{Na}}^{\text{surface}} g_{\text{Na}}^{\text{bulk}} + n_{\text{K}}^{\text{surface}} g_{\text{K}}^{\text{bulk}} + \sigma (n_{\text{Na}}^{\text{surface}} A_{\text{Na}} + n_{\text{K}}^{\text{surface}} A_{\text{K}}) \\ &= n_{\text{Na}}^{\text{surface}} (g_{\text{Na}}^{\text{bulk}} + \sigma A_{\text{Na}}) + n_{\text{K}}^{\text{surface}} (g_{\text{K}}^{\text{bulk}} + \sigma A_{\text{K}}) \\ &= n_{\text{Na}}^{\text{surface}} (g_{\text{Na}}^{\text{bulk,pure}} + A_{\text{Na}}^{\text{pure}} \sigma_{\text{Na}} + RT \ln a_{\text{Na}}^{\text{surface}}) + n_{\text{K}}^{\text{surface}} (g_{\text{K}}^{\text{bulk,pure}} + A_{\text{K}}^{\text{pure}} \sigma_{\text{K}} + RT \ln a_{\text{K}}^{\text{surface}}) \\ &= n_{\text{Na}}^{\text{surface}} (g_{\text{Na}}^{\text{bulk,pure}} + A_{\text{Na}}^{\text{pure}} \sigma_{\text{Na}} + RT \ln x_{\text{Na}}^{\text{surface}} + \beta \cdot G_{\text{Na,}}^{\text{E,bulk}}(T, x_{\text{Na}}^{\text{surface}})) + \\ &\quad n_{\text{K}}^{\text{surface}} (g_{\text{K}}^{\text{bulk,pure}} + A_{\text{K}}^{\text{pure}} \sigma_{\text{K}} + RT \ln x_{\text{K}}^{\text{surface}} + \beta \cdot G_{\text{K}}^{\text{E,bulk}}(T, x_{\text{Na}}^{\text{surface}})) \end{aligned} \quad (20)$$

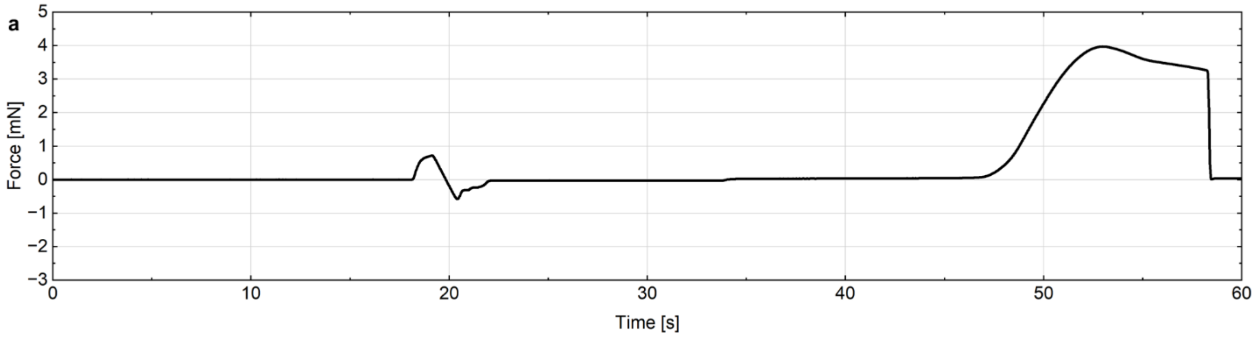

Figure S1. Du Noüy ring measurements of silicone oil. a, Force acting on the Du Noüy ring versus time during a representative measurement with silicone oil at room temperature.

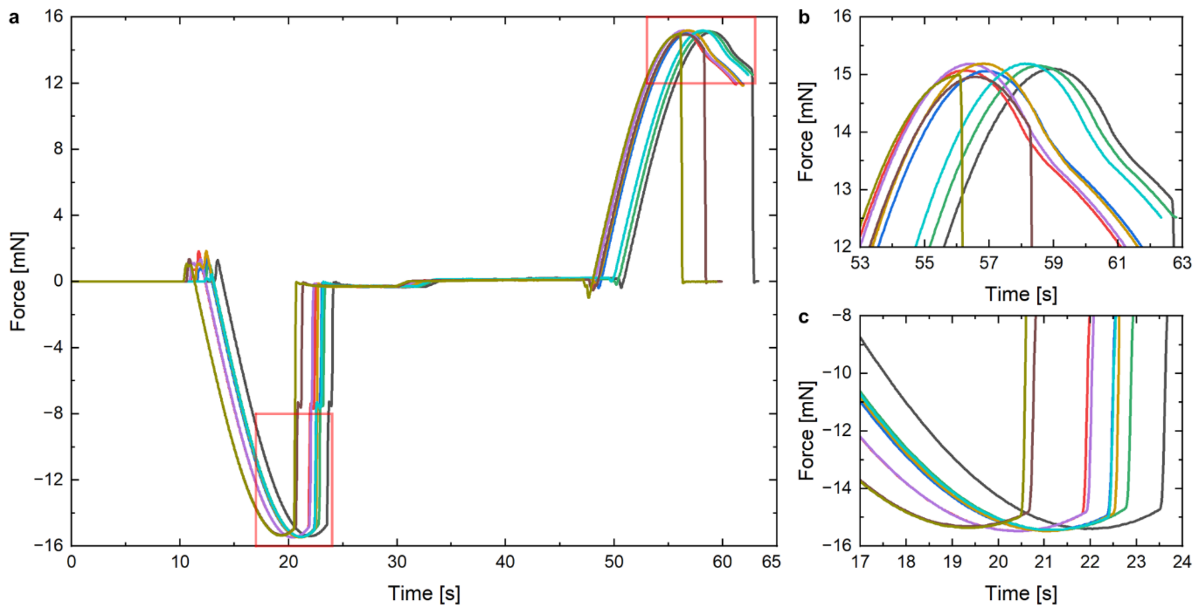

Figure S2. Raw data for $Na_{68}K_{32}$ measurements at room temperature. a, Raw data for the nine measurements at room temperature. **b**, A zoom-in view focused on the maximum force. **c**, A zoom-in view focused on the minimum force.

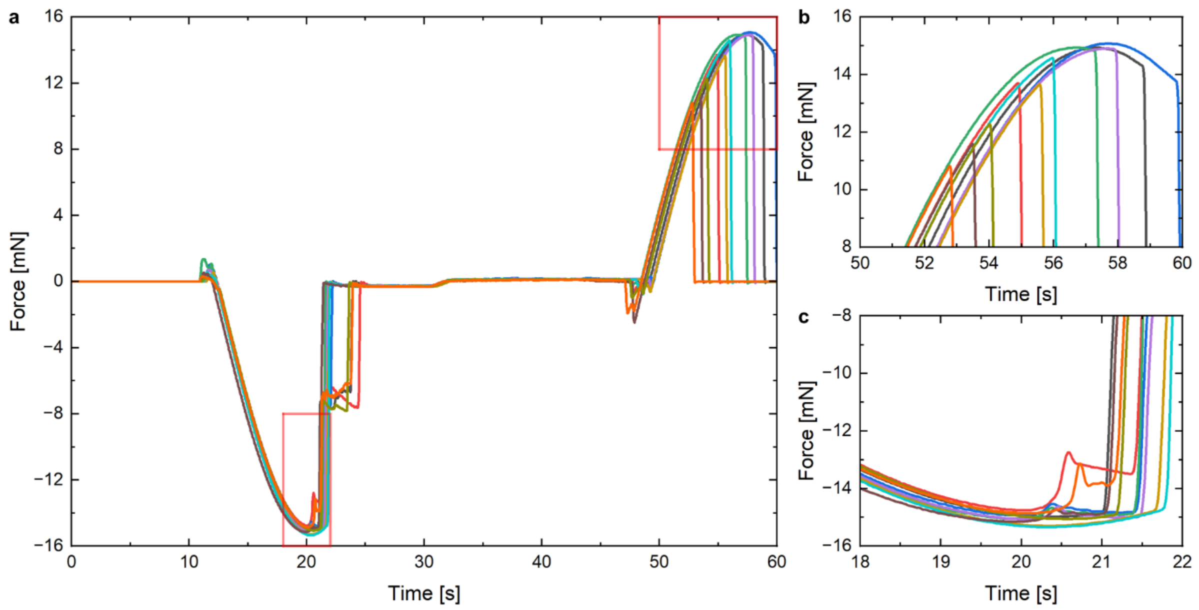

Figure S3. Raw data for $Na_{68}K_{32}$ measurements at 70°C. a, Raw data for the ten measurements at 70°C. **b**, A zoom-in view focused on the maximum force. **c**, A zoom-in view focused on the minimum force.

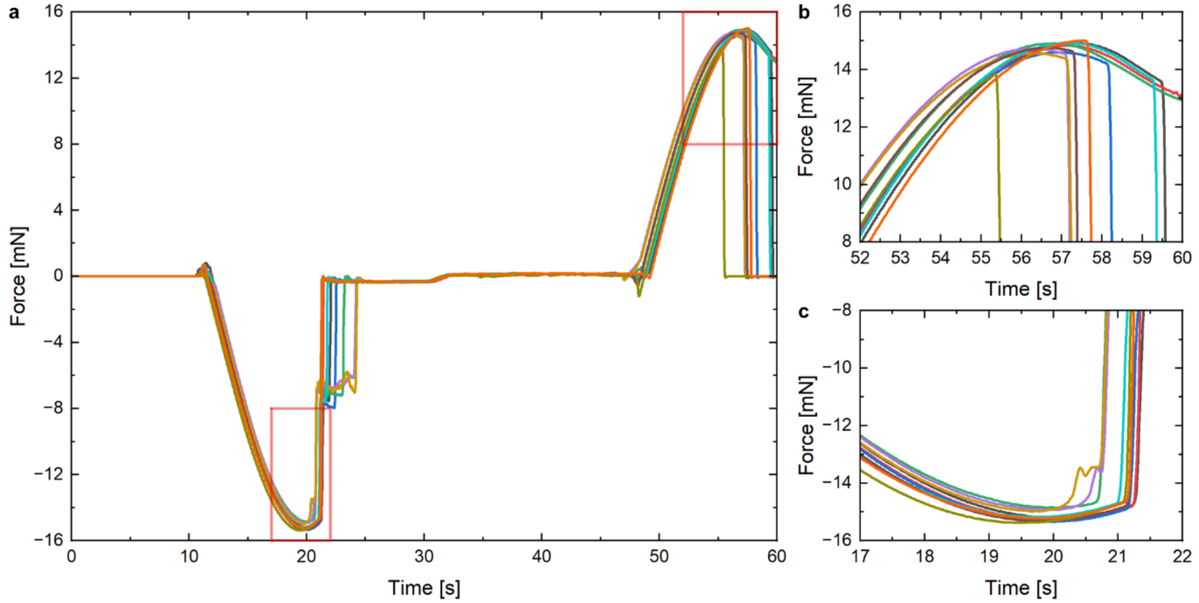

Figure S4. Raw data for $Na_{68}K_{32}$ measurements at 110°C. a, Raw data for the eleven measurements at 110°C. **b**, A zoom-in view focused on the maximum force. **c**, A zoom-in view focused on the minimum force.

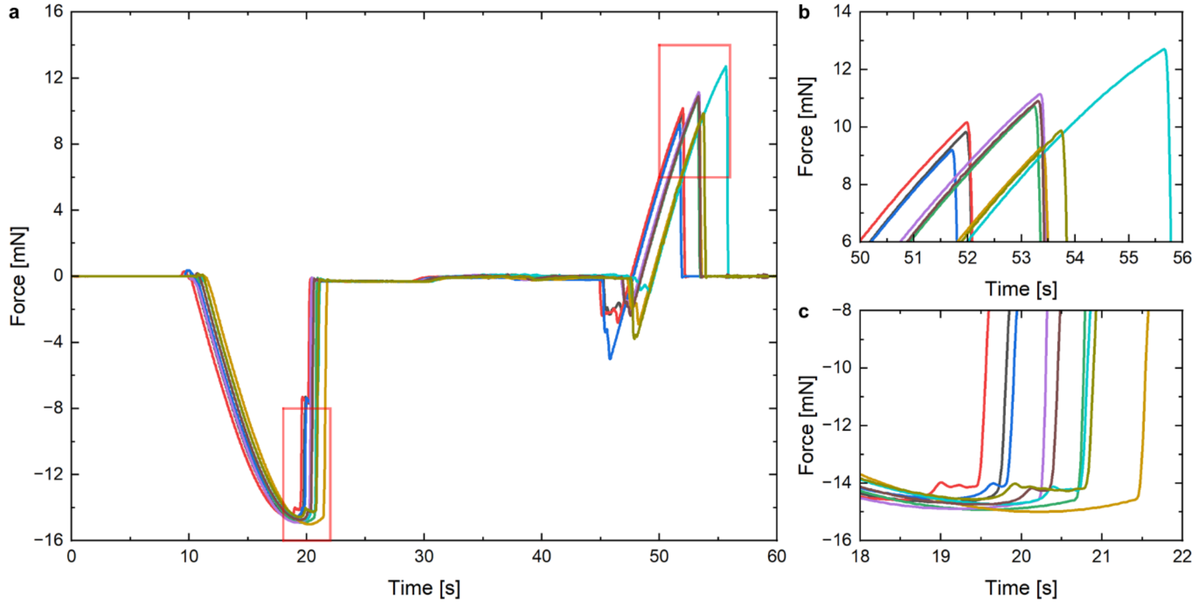

Figure S5. Raw data for $Na_{68}K_{32}$ measurements at 150°C. a, Raw data for the nine measurements at 150°C. **b**, A zoom-in view focused on the maximum force. **c**, A zoom-in view focused on the minimum force.

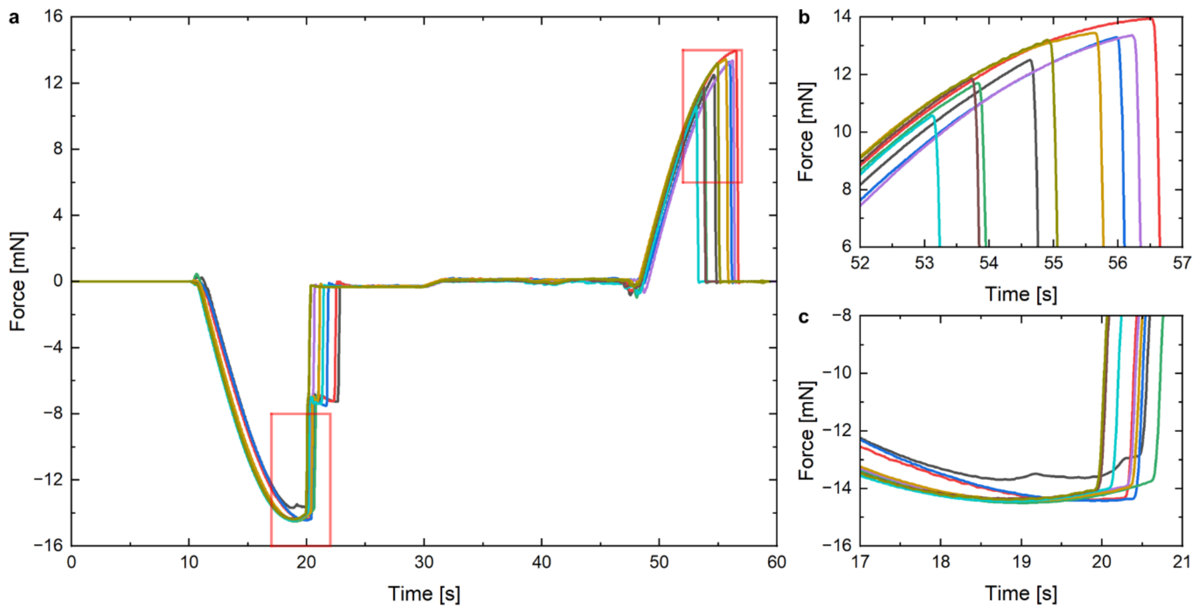

Figure S6. Raw data for $Na_{68}K_{32}$ measurements at 180°C. a, Raw data for the nine measurements at 180°C. **b**, A zoom-in view focused on the maximum force. **c**, A zoom-in view focused on the minimum force.

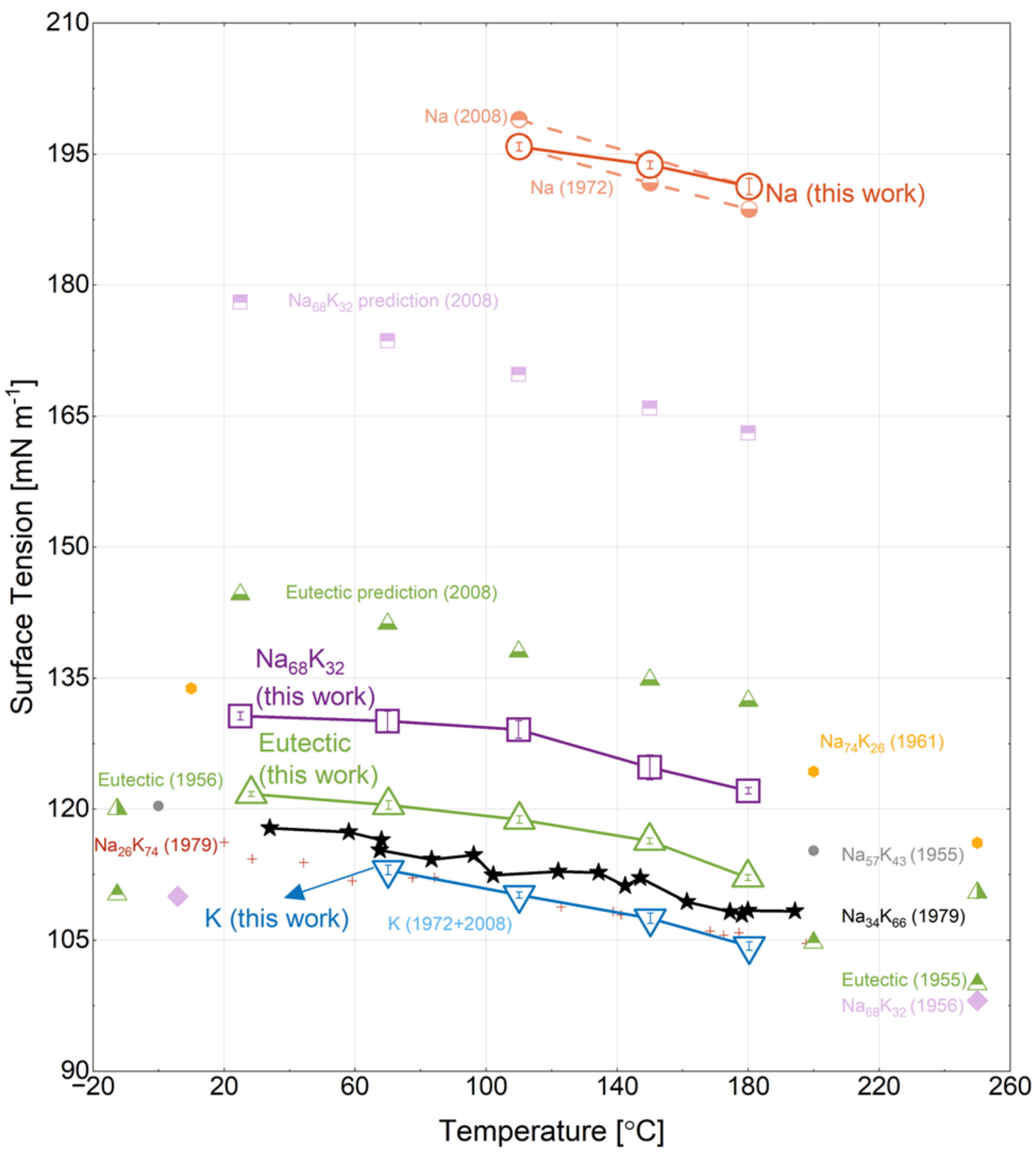

Figure S7. Comparison between existing values in literature and measured values in this work for the Na-K system.[5–10]

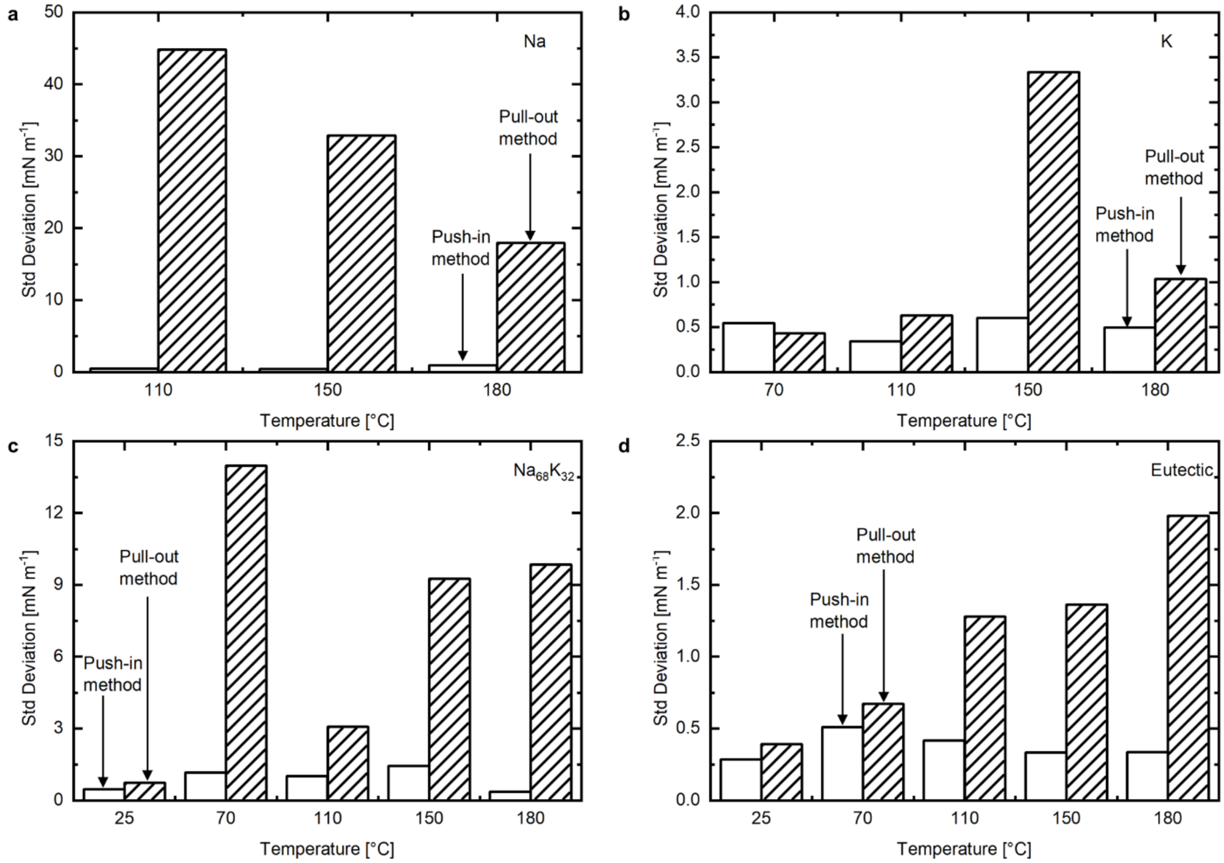

Figure S8. Standard deviations of push-in and pull-out methods for four different compositions studied in our work. a, Standard deviations of push-in and pull-out methods for Na measurements across temperatures. **b,** Standard deviations of push-in and pull-out methods for K measurements across temperatures. **c,** Standard deviations of push-in and pull-out methods for $Na_{68}K_{32}$ measurements across temperatures. **d,** Standard deviations of push-in and pull-out methods for eutectic Na-K alloy measurements across temperatures.

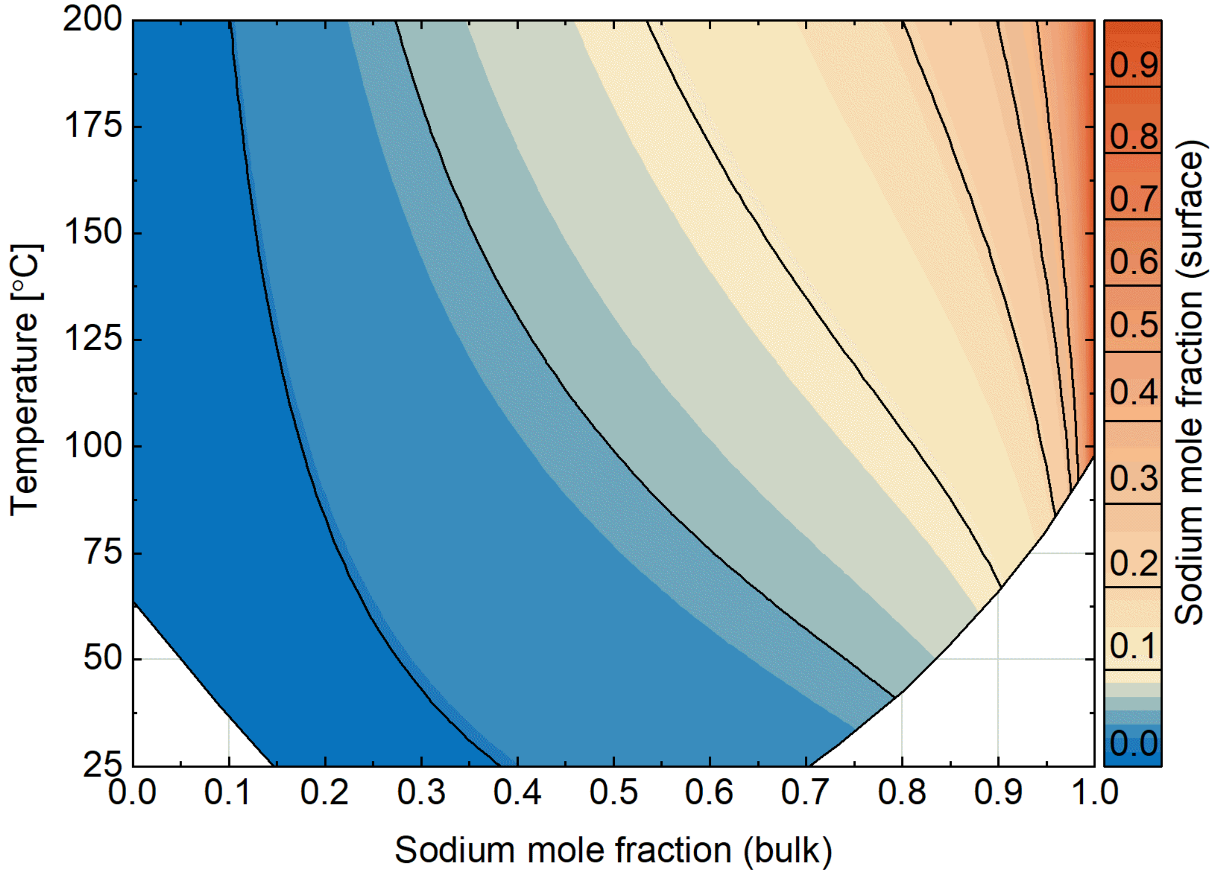

Figure S9. Contour plot illustrating the variation of sodium mole fraction in the surface phase of the NaK alloy as a function of sodium mole fraction in the bulk phase and temperature (°C).

Table S1. The detailed data for the Du noüy ring measurements measured in this work

Na			$Na_{68}K_{32}$		
**Temperature**	**Surface Tension**	**Standard Deviation**	**Temperature**	**Surface Tension**	**Standard Deviation**
°C	mN m^{-1}	mN m^{-1}	°C	mN m^{-1}	mN m^{-1}
110.1	195.8	0.5	25.0	130.6	0.5
150.1	193.7	0.4	70.0	130.0	1.2
180.3	191.3	0.9	109.9	129.1	1.0
			150.0	124.8	1.4
			180.1	122.1	0.4
**K**			**Eutectic**		
**Temperature**	**Surface Tension**	**Standard Deviation**	**Temperature**	**Surface Tension**	**Standard Deviation**
°C	mN m^{-1}	mN m^{-1}	°C	mN m^{-1}	mN m^{-1}
70.1	113.0	0.5	28.3	121.7	0.3
110.1	110.2	0.3	70.2	120.5	0.5
150.2	107.5	0.6	110.2	118.8	0.4
180.3	104.3	0.5	149.9	116.4	0.3
			180.0	112.2	0.3

Table S2. The detailed data for averaged standard deviations in the push-in and pull-out methods across temperatures.

	Na	K	$Na_{68}K_{32}$	Eutectic	Average Std Devi
Temperature	Push-in Method				
	Standard Deviation (mN m^{-1})				Average Std Devi (mN m^{-1})
°C					
25			0.5	0.3	0.4
70		0.5	1.2	0.5	0.7
110	0.5	0.3	1.0	0.4	0.6
150	0.4	0.6	1.4	0.3	0.7
180	0.9	0.5	0.4	0.3	0.5
Temperature	Pull-out method				
	Standard Deviation (mN m^{-1})				Average Std Devi (mN m^{-1})
°C					
25			0.7	0.4	0.6
70		0.4	14.0	0.7	5.1
110	44.8	0.6	3.1	1.3	12.4
150	32.9	3.3	9.3	1.4	11.7
180	17.9	1.0	9.8	2.0	7.7

Table S3. The detailed comparison between the calculated and measured surface tension of two Na-K alloys.

	Temperature	Measured	Calculated	Difference	Deviation
	°C	mN m^{-1}	mN m^{-1}	mN m^{-1}	%
$Na_{68}K_{32}$	25	130.6	126.8	-3.8	-2.9
	70	130.0	126.7	-3.3	-2.5
	110	129.1	126.4	-2.7	-2.1
	150	124.8	125.9	1.1	0.9
	180	122.1	125.3	3.2	2.6
Eutectic	25	121.7	119.6	-2.1	-1.8
	70	120.5	117.6	-2.8	-2.3
	110	118.8	115.7	-3.1	-2.6
	150	116.4	113.7	-2.6	-2.3
	180	112.2	113.7	1.6	1.4

Table S4. The isothermal change of surface tension and sodium mole fraction in the surface phase at 110 °C, as compared to the sodium mole fraction in the bulk phase.

Sodium mole fraction (bulk)	Modeled surface tension	Sodium mole fraction (surface)	Measured surface tension
	mN m^{-1}		mN m^{-1}
**0.00**	108.7	0.0000	110.2
**0.01**	108.9	0.0013	
**0.02**	109.1	0.0027	
**0.03**	109.3	0.0040	
**0.04**	109.5	0.0053	
**0.05**	109.8	0.0066	
**0.06**	110.0	0.0078	
**0.07**	110.2	0.0091	
**0.08**	110.4	0.0103	
**0.09**	110.7	0.0115	
**0.10**	110.9	0.0127	
**0.11**	111.1	0.0139	
**0.12**	111.3	0.0151	
**0.13**	111.5	0.0162	
**0.14**	111.7	0.0174	
**0.15**	112.0	0.0185	
**0.16**	112.2	0.0196	
**0.17**	112.4	0.0207	
**0.18**	112.6	0.0218	
**0.19**	112.8	0.0229	
**0.20**	113.0	0.0239	
**0.21**	113.2	0.0250	
**0.22**	113.5	0.0260	
**0.23**	113.7	0.0270	
**0.24**	113.9	0.0280	
**0.25**	114.1	0.0290	
**0.26**	114.3	0.0300	
**0.27**	114.5	0.0310	
**0.28**	114.8	0.0320	
**0.29**	115.0	0.0330	
**0.30**	115.2	0.0339	
**0.31**	115.4	0.0349	
**0.32**	115.6	0.0359	
**0.324**	115.7	0.0363	118.8

**0.33**	115.9	0.0368	
**0.34**	116.1	0.0378	
**0.35**	116.3	0.0388	
**0.36**	116.6	0.0398	
**0.37**	116.8	0.0407	
**0.38**	117.0	0.0417	
**0.39**	117.3	0.0427	
**0.40**	117.5	0.0437	
**0.41**	117.8	0.0447	
**0.42**	118.0	0.0457	
**0.43**	118.3	0.0468	
**0.44**	118.5	0.0478	
**0.45**	118.8	0.0488	
**0.46**	119.1	0.0499	
**0.47**	119.3	0.0510	
**0.48**	119.6	0.0521	
**0.49**	119.9	0.0532	
**0.50**	120.2	0.0543	
**0.51**	120.5	0.0554	
**0.52**	120.8	0.0566	
**0.53**	121.1	0.0578	
**0.54**	121.4	0.0590	
**0.55**	121.7	0.0602	
**0.56**	122.0	0.0614	
**0.57**	122.4	0.0627	
**0.58**	122.7	0.0640	
**0.59**	123.1	0.0653	
**0.60**	123.4	0.0667	
**0.61**	123.8	0.0681	
**0.62**	124.2	0.0695	
**0.63**	124.6	0.0710	
**0.64**	125.0	0.0725	
**0.65**	125.4	0.0740	
**0.66**	125.8	0.0756	
**0.67**	126.2	0.0773	
**0.675**	126.4	0.0781	129.1
**0.68**	126.7	0.0790	
**0.69**	127.1	0.0807	
**0.70**	127.6	0.0825	
**0.71**	128.1	0.0844	
**0.72**	128.6	0.0864	

**0.73**	129.1	0.0884	
**0.74**	129.6	0.0905	
**0.75**	130.2	0.0927	
**0.76**	130.7	0.0950	
**0.77**	131.3	0.0975	
**0.78**	132.0	0.1000	
**0.79**	132.6	0.1028	
**0.80**	133.3	0.1057	
**0.81**	134.1	0.1088	
**0.82**	134.8	0.1121	
**0.83**	135.7	0.1158	
**0.84**	136.6	0.1197	
**0.85**	137.5	0.1240	
**0.86**	138.5	0.1288	
**0.87**	139.6	0.1341	
**0.88**	140.9	0.1402	
**0.89**	142.2	0.1471	
**0.90**	143.7	0.1551	
**0.91**	145.4	0.1645	
**0.92**	147.3	0.1760	
**0.93**	149.5	0.1901	
**0.94**	152.1	0.2083	
**0.95**	155.2	0.2325	
**0.96**	159.1	0.2667	
**0.97**	164.1	0.3192	
**0.98**	170.8	0.4095	
**0.99**	180.9	0.5942	
**1.00**	195.7	1.0000	195.8

Table S5. Selected calculated surface tension for the Na-K system, as compared to the sodium mole fraction in the bulk phase, x_{Na}.

	Temperature (°C)								
x_{Na}	**25**	**40**	**50**	**60**	**70**	**80**	**90**	**100**	**110**
**0.00**	114.1	113.1	112.5	111.9	111.2	110.6	109.9	109.3	108.7
**0.05**	115.0	114.1	113.5	112.9	112.3	111.6	111.0	110.4	109.8
**0.10**	116.0	115.1	114.5	113.9	113.3	112.7	112.1	111.5	110.9
**0.15**	116.8	116.0	115.4	114.8	114.3	113.7	113.1	112.5	112.0
**0.20**	117.7	116.9	116.3	115.8	115.3	114.7	114.1	113.6	113.0
**0.25**	118.5	117.8	117.3	116.7	116.2	115.7	115.2	114.6	114.1
**0.30**	119.3	118.6	118.2	117.7	117.2	116.7	116.2	115.7	115.2
**0.35**	120.2	119.5	119.1	118.6	118.2	117.7	117.3	116.8	116.3
**0.40**	121.0	120.4	120.0	119.6	119.2	118.8	118.4	117.9	117.5
**0.45**	121.9	121.4	121.0	120.7	120.3	119.9	119.6	119.2	118.8
**0.50**	122.8	122.4	122.1	121.8	121.5	121.2	120.8	120.5	120.2
**0.55**	123.8	123.5	123.3	123.0	122.8	122.5	122.3	122.0	121.7
**0.60**	124.9	124.7	124.5	124.4	124.2	124.0	123.9	123.6	123.4
**0.65**	126.1	126.1	126.0	125.9	125.8	125.7	125.6	125.5	125.4
**0.70**	127.5	127.6	127.7	127.7	127.7	127.7	127.7	127.6	127.6
**0.75**	129.2	129.5	129.6	129.8	129.9	130.0	130.1	130.1	130.2
**0.80**	131.3	131.8	132.1	132.3	132.6	132.8	133.0	133.2	133.3
**0.85**	134.2	134.9	135.4	135.8	136.2	136.6	136.9	137.2	137.5
**0.90**	138.8	139.9	140.5	141.2	141.7	142.3	142.8	143.3	143.7
**0.95**	148.2	149.8	150.7	151.6	152.5	153.2	154.0	154.6	155.2
**1.00**	204.2	202.7	201.7	200.7	199.7	198.7	197.7	196.7	195.7

	Temperature (°C)								
x_{Na}	**120**	**130**	**140**	**150**	**160**	**170**	**180**	**190**	**200**
**0.00**	108.0	107.4	106.7	106.1	105.5	104.8	104.2	103.5	102.9
**0.05**	109.1	108.5	107.9	107.3	106.7	106.0	105.4	104.8	104.1
**0.10**	110.3	109.7	109.1	108.4	107.8	107.2	106.6	106.0	105.4
**0.15**	111.4	110.8	110.2	109.6	109.0	108.4	107.8	107.2	106.6
**0.20**	112.5	111.9	111.3	110.8	110.2	109.6	109.0	108.5	107.9
**0.25**	113.6	113.0	112.5	111.9	111.4	110.8	110.3	109.7	109.2
**0.30**	114.7	114.2	113.7	113.1	112.6	112.1	111.5	111.0	110.5
**0.35**	115.8	115.4	114.9	114.4	113.9	113.4	112.9	112.4	111.9
**0.40**	117.1	116.6	116.2	115.7	115.3	114.8	114.3	113.8	113.3
**0.45**	118.4	118.0	117.6	117.2	116.7	116.3	115.9	115.4	115.0
**0.50**	119.8	119.5	119.1	118.7	118.4	118.0	117.6	117.2	116.7
**0.55**	121.4	121.1	120.8	120.5	120.2	119.8	119.5	119.1	118.7
**0.60**	123.2	123.0	122.7	122.4	122.2	121.9	121.6	121.3	120.9
**0.65**	125.2	125.0	124.9	124.7	124.4	124.2	124.0	123.7	123.4
**0.70**	127.5	127.4	127.3	127.2	127.0	126.9	126.7	126.5	126.3
**0.75**	130.2	130.2	130.2	130.1	130.1	130.0	129.9	129.7	129.6
**0.80**	133.5	133.6	133.6	133.7	133.7	133.7	133.7	133.6	133.6
**0.85**	137.7	138.0	138.1	138.3	138.4	138.5	138.6	138.6	138.6
**0.90**	144.1	144.4	144.7	145.0	145.2	145.4	145.5	145.6	145.7
**0.95**	155.8	156.2	156.6	157.0	157.2	157.4	157.6	157.6	157.7
**1.00**	194.7	193.7	192.7	191.7	190.7	189.7	188.7	187.7	186.7

Table S6. Selected calculated sodium mole fraction in the surface phase for the Na-K system, as compared to the sodium mole fraction in the bulk phase, x_{Na}.

	Temperature (°C)								
x_{Na}	25	40	50	60	70	80	90	100	110
**0.00**	0.0000	0.0000	0.0000	0.0000	0.0000	0.0000	0.0000	0.0000	0.0000
**0.05**	0.0034	0.0040	0.0043	0.0047	0.0050	0.0054	0.0058	0.0062	0.0066
**0.10**	0.0066	0.0076	0.0083	0.0090	0.0097	0.0105	0.0112	0.0120	0.0127
**0.15**	0.0095	0.0110	0.0120	0.0130	0.0141	0.0152	0.0163	0.0174	0.0185
**0.20**	0.0121	0.0140	0.0154	0.0167	0.0181	0.0195	0.0210	0.0224	0.0239
**0.25**	0.0145	0.0168	0.0185	0.0201	0.0218	0.0236	0.0254	0.0272	0.0290
**0.30**	0.0167	0.0194	0.0214	0.0233	0.0254	0.0275	0.0296	0.0317	0.0339
**0.35**	0.0187	0.0219	0.0241	0.0264	0.0288	0.0312	0.0337	0.0362	0.0388
**0.40**	0.0207	0.0243	0.0268	0.0295	0.0322	0.0350	0.0378	0.0407	0.0437
**0.45**	0.0226	0.0267	0.0296	0.0326	0.0356	0.0388	0.0421	0.0454	0.0488
**0.50**	0.0246	0.0292	0.0324	0.0358	0.0393	0.0429	0.0466	0.0504	0.0543
**0.55**	0.0266	0.0318	0.0354	0.0392	0.0431	0.0472	0.0514	0.0557	0.0602
**0.60**	0.0288	0.0345	0.0386	0.0429	0.0473	0.0519	0.0567	0.0616	0.0667
**0.65**	0.0312	0.0376	0.0421	0.0469	0.0519	0.0572	0.0626	0.0682	0.0740
**0.70**	0.0338	0.0410	0.0461	0.0516	0.0573	0.0632	0.0694	0.0759	0.0825
**0.75**	0.0370	0.0450	0.0509	0.0570	0.0636	0.0704	0.0775	0.0850	0.0927
**0.80**	0.0409	0.0501	0.0569	0.0640	0.0716	0.0795	0.0879	0.0966	0.1057
**0.85**	0.0465	0.0574	0.0653	0.0738	0.0828	0.0924	0.1024	0.1130	0.1240
**0.90**	0.0561	0.0697	0.0797	0.0905	0.1020	0.1142	0.1272	0.1408	0.1551
**0.95**	0.0803	0.1007	0.1159	0.1324	0.1501	0.1691	0.1892	0.2103	0.2325
**1.00**	1.0000	1.0000	1.0000	1.0000	1.0000	1.0000	1.0000	1.0000	1.0000

	Temperature (°C)								
x_{Na}	**120**	**130**	**140**	**150**	**160**	**170**	**180**	**190**	**200**
**0.00**	0.0000	0.0000	0.0000	0.0000	0.0000	0.0000	0.0000	0.0000	0.0000
**0.05**	0.0069	0.0073	0.0077	0.0081	0.0085	0.0089	0.0093	0.0097	0.0101
**0.10**	0.0135	0.0143	0.0150	0.0158	0.0166	0.0174	0.0182	0.0189	0.0197
**0.15**	0.0196	0.0208	0.0219	0.0231	0.0242	0.0254	0.0266	0.0277	0.0289
**0.20**	0.0254	0.0269	0.0284	0.0300	0.0315	0.0330	0.0346	0.0361	0.0376
**0.25**	0.0309	0.0327	0.0346	0.0365	0.0384	0.0404	0.0423	0.0442	0.0461
**0.30**	0.0362	0.0384	0.0407	0.0430	0.0453	0.0476	0.0499	0.0522	0.0546
**0.35**	0.0414	0.0440	0.0467	0.0494	0.0521	0.0549	0.0576	0.0604	0.0632
**0.40**	0.0467	0.0498	0.0529	0.0561	0.0592	0.0624	0.0656	0.0689	0.0721
**0.45**	0.0523	0.0558	0.0594	0.0631	0.0667	0.0704	0.0742	0.0779	0.0817
**0.50**	0.0583	0.0623	0.0664	0.0706	0.0748	0.0791	0.0834	0.0878	0.0922
**0.55**	0.0647	0.0694	0.0741	0.0789	0.0838	0.0887	0.0937	0.0988	0.1038
**0.60**	0.0719	0.0772	0.0827	0.0882	0.0938	0.0995	0.1053	0.1112	0.1171
**0.65**	0.0800	0.0861	0.0924	0.0988	0.1053	0.1119	0.1186	0.1254	0.1323
**0.70**	0.0894	0.0965	0.1037	0.1111	0.1187	0.1264	0.1342	0.1422	0.1502
**0.75**	0.1007	0.1089	0.1174	0.1261	0.1349	0.1440	0.1532	0.1625	0.1720
**0.80**	0.1151	0.1248	0.1349	0.1452	0.1558	0.1665	0.1775	0.1886	0.1999
**0.85**	0.1355	0.1474	0.1596	0.1722	0.1851	0.1983	0.2117	0.2253	0.2390
**0.90**	0.1700	0.1854	0.2013	0.2177	0.2344	0.2514	0.2685	0.2857	0.3028
**0.95**	0.2554	0.2789	0.3027	0.3267	0.3505	0.3741	0.3971	0.4194	0.4410
**1.00**	1.0000	1.0000	1.0000	1.0000	1.0000	1.0000	1.0000	1.0000	1.0000

Reference

(1) Kang, Y.-B. Relationship between Surface Tension and Gibbs Energy, and Application of Constrained Gibbs Energy Minimization. *Calphad* **2015**, *50*, 23–31. https://doi.org/10.1016/j.calphad.2015.04.008.

(2) Butler, J. A. V. The Thermodynamics of the Surfaces of Solutions. *Proc. R. Soc. Lond. Ser. A* **1932**, *135* (827). https://doi.org/10.1098/rspa.1932.0040.

(3) Tanaka, T.; Matsuda, M.; Nakao, K.; Katayama, Y.; Kaneko, D.; Hara, S.; Xing, X.; Qiao, Z. Measurement of Surface Tension of Liquid Ga-Base Alloys by a Sessile Drop Method. *Int. J. Mater. Res.* **2001**, *92* (11), 1242–1246. https://doi.org/10.3139/ijmr-2001-0228.

(4) Bale, C. W. The K−Na (Potassium−Sodium) System. *Bull. Alloy Phase Diagr.* **1982**, *3* (3), 313–318. https://doi.org/10.1007/BF02869301.

(5) Foust, O. J. *SODIUM-NaK ENGINEERING HANDBOOK. VOLUME I. SODIUM CHEMISTRY AND PHYSICAL PROPERTIES.*; TID-26008; 1972, 1972. https://www.osti.gov/biblio/4631555.

(6) *Thermophysical Properties of Materials for Nuclear Engineering: A Tutorial and Collection of Data*; International Atomic Energy Agency, 2008.

(7) Sittig, M. *Sodium: Its Manufacture, Properties, and Uses*; New York, Reinhold Pub. Corp., 1956.

(8) Jackson, C. B. *Liquid-Metals Handbook: Sodium-NaK Supplement*; Atomic Energy Commission, 1955.

(9) Defense Technical Information Center. *DTIC ADA073128: Experimental Two-Phase Liquid-Metal Magnetohydrodynamic Generator Program*; 1979.

(10) Bradhurst, D. H.; Buchanan, A. S. Surface Properties of Liquid Sodium and Sodium-Potassium Alloys in Contact with Metal Oxide Surfaces. *Aust. J. Chem.* **1961**, *14* (3). https://doi.org/10.1071/CH9610397.
